# Induction of m^6^A methylation in adipocyte exosomal LncRNAs mediates myeloma drug resistance

**DOI:** 10.1186/s13046-021-02209-w

**Published:** 2022-01-03

**Authors:** Zhiming Wang, Jin He, Duc-hiep Bach, Yung-hsing Huang, Zongwei Li, Huan Liu, Pei Lin, Jing Yang

**Affiliations:** 1 Houston Methodist Cancer Center, Research Institute Houston Methodist Hospital, Houston, TX 77030 USA; 2 Cancer Research Center, School of Medicine, Xiamen University, Xiamen, 361102 China; 3 Department of Hematopathology, The University of Texas MD Anderson Cancer Center, Houston, TX 77030 USA

**Keywords:** Myeloma, Adipocytes, Exosomes, LncRNA m^6^A Methylation, Therapeutics

## Abstract

**Background:**

Therapeutic resistance occurs in most patients with multiple myeloma (MM). One of the key mechanisms for MM drug resistance comes from the interaction between MM cells and adipocytes that inhibits drug-induced apoptosis in MM cells; MM cells reprogram adipocytes to morph into different characterizations, including exosomes, which are important for tumor-stroma cellular communication. However, the mechanism by which exosomes mediate the cellular machinery of the vicious cycle between MM cells and adipocytes remains unclear.

**Methods:**

Adipocytes were either isolated from bone marrow aspirates of healthy donors or MM patients or derived from mesenchymal stem cells. Co-culturing normal adipocytes with MM cells was used to generate MM-associated adipocytes. Exosomes were collected from the culture medium of adipocytes. Annexin V-binding and TUNEL assays were performed to assess MM cell apoptosis. Methyltransferase activity assay and dot blotting were used to access the m^6^A methylation activity of methyltransferase like 7A (METTL7A). RIP, MeRIP-seq, and RNA–protein pull down for assessing the interaction between long non-cording RNAs (LncRNAs) and RNA binding proteins were performed. Adipocyte-specific enhancer of zeste homolog 2 (EZH2) knockout mice and MM-xenografted mice were used for evaluating MM therapeutic response in vivo.

**Results:**

Exosomes collected from MM patient adipocytes protect MM cells from chemotherapy-induced apoptosis. Two LncRNAs in particular, LOC606724 and SNHG1, are significantly upregulated in MM cells after exposure to adipocyte exosomes. The raised LncRNA levels in MM cells are positively correlated to worse outcomes in patients, indicating their clinical relevancy in MM. The functional roles of adipocyte exosomal LOC606724 or SNHG1 in inhibition of MM cell apoptosis are determined by knockdown in adipocytes or overexpression in MM cells. We discovered the interactions between LncRNAs and RNA binding proteins and identified methyltransferase like 7A (METTL7A) as an RNA methyltransferase. MM cells promote LncRNA package into adipocyte exosomes through METTL7A-mediated LncRNA m^6^A methylation. Exposure of adipocytes to MM cells enhances METTL7A activity in m^6^A methylation through EZH2-mediated protein methylation.

**Conclusion:**

This study elucidates an unexplored mechanism of how adipocyte-rich microenvironment exacerbates MM therapeutic resistance and indicates a potential strategy to improve therapeutic efficacy by blocking this vicious exosome-mediated cycle.

**Supplementary Information:**

The online version contains supplementary material available at 10.1186/s13046-021-02209-w.

## Background

Multiple myeloma (MM) is a malignancy of antibody producing plasma cells that accumulate in bone marrow [1, 2]. It is the second most common hematological malignancy in the United States and has been estimated to account for more than 10% of all hematological malignancies [3]. Current therapeutic agents, such as proteasome inhibitors bortezomib or carfilzomib, offer remarkable benefits for MM patients [4]. However, most MM patients go into relapse or refractory disease, making drug resistance the major roadblock for a cure [5]. Therefore, understanding the molecular mechanism underlying MM drug resistance will be key to improving the efficacy of MM therapeutic drugs and prolonging patient survival.

As one of the most abundant stromal cells within the bone marrow where MM cells reside [6, 7], adipocytes have been shown to participate in the development and pathogenesis of MM and not just for the sole function of providing energy to cells [8–10]. Adipocytes can promote MM growth, mediate obesity-induced tumorigenesis, recruit tumor cells to specific bone area, and regulate osteoclast and osteoblast differentiation and activity [8, 11, 12]. Notably, marrow adipocytes contribute to MM drug resistance through inhibition of chemotherapy-induced tumor cell apoptosis [13, 14]. While studies have shown that a number of adipokines secreted from adipocytes are responsible for the protective effect against MM treatment [13–15], other adipocyte-derived factors, such as extracellular vesicles, have yet to be investigated.

MM cells, on the other hand, manipulate the host bone marrow to make it be a more conducive microenvironment for MM cell growth and survival, thereby forming a vicious cycle with surrounding stromal cells [16]. For example, adipocytes can be reprogrammed by MM cells [12] and morphed into different profiles that display different characteristics. We previously found that MM cells activate histone methylation in the promoter of adipokine genes through upregulation of enhancer of zeste homolog 2 (EZH2) expression in adipocytes, and the reprogrammed adipocytes secret a different set of adipokines/cytokines [12]. Other studies also found that MM cells reduce adipocytic gene expression and induce senescence and metabolic changes in adipocytes [10]. However, there is little knowledge of the effect of MM cells on the adipocyte production of exosomes.

Exosomes are nanometer-sized extracellular vesicles shown to be important for tumor-stroma cellular communication and mediation of stroma-induced tumor drug resistance. They originate from multivesicular bodies whose membrane invaginates to form intraluminal vesicles [17]. Non-coding RNAs, including long non-coding RNAs (LncRNAs), can be packed and transferred into the vesicles through RNA binding proteins [17]. Interestingly, the loading of exosomal RNAs is highly selective, and differential expression of exosomal RNAs may be associated with different biological functions [18]. In addition, RNAs with relatively low levels of cellular expression have been found to be highly enriched in secreted exosomes [19], while the biogenesis of such selection or abundance has yet to be elucidated. In this study, we hypothesized that MM cells may modulate LncRNA enrichment into adipocyte exosomes, and in turn, adipocyte-derived exosomal LncRNAs inhibit chemotherapy-induced apoptosis in MM cells. The goal of this study was to unveil the cellular machinery of a vicious cycle between MM cells and adipocytes, and thus shed light on an unexplored mechanism of bone marrow microenvironment-induced MM drug resistance.

## Methods

### Cell lines and primary MM cells

The MM cell line ARP-1 was provided by the University of Arkansas for Medical Sciences. Murine MM Vk*MYC cell line (Vk12598) was provided by the Mayo Clinic [20]. HEK293T, MM.1S, U266, and RPMI8226 cells were purchased from the American Type Culture Collection. Primary MM cells were isolated from bone marrow aspirates from patients with MM using anti-CD138 antibody-coated magnetic beads (Miltenyi Biotec, Germany). MM cells were maintained in RPMI 1640 medium with 10% fetal bovine serum (FBS), and HEK293T cells were cultured in Dulbecco’s modified Eagle’s medium (DMEM) with 10% FBS. All patient samples were obtained from the Biorepository of Houston Methodist Research Institute (HMRI) or the Myeloma Tissue Bank of UT MD Anderson Cancer Center (MDACC). This study was approved by the Institutional Review Board of HMRI and MDACC. In some experiments, MM cells were cultured with 20 μg exosomes/10^5^ cells or treated with chemotherapeutic drugs, such as bortezomib (5 nM), melphalan (25 μM), or carfilzomib (25 nM).

### Antibodies and reagents

Except where specified, all chemicals were purchased from Sigma-Aldrich (St. Louis, MO) or Caymen Chemical Company (Ann Arbor, MI), all antibodies for flow cytometric analysis were purchased from BD Biosciences (Franklin Lakes, NJ), all ELISA kits were purchased from R&D Systems (Minneapolis, MN), and all antibodies for Western blot analysis were purchased from Cell Signaling Technology (Danvers, MA). Tamoxifen was obtained from Sigma-Aldrich and dissolved in corn oil at a concentration of 20 mg/ml as recommended by the manufacturer.

### ORFs, shRNAs, RNA oligonucleotides, and siRNAs

*LOC606724* or *SNHG1* were sub-cloned into a pcDNA3.1 vector, and the related primers are listed in Table S1. ORF plasmid for c-Myc overexpression was purchased from OriGene, Rockville, MD. Full length and truncated forms of METTL7A were sub-cloned into pET28a vector (EMD Millipore, Burlington, MA), and their respective form of His-tagged proteins was expressed and purified according to the manufacturer protocol; related primers are listed in Table S1. sh*Ctrl*, sh*Loc*, and sh*SNHG1* were sub-cloned into pLKO.1 vector; related primers are listed in Table S2. Custom RNA oligonucleotides containing various putative METTL7A binding site on *LOC* transcript are listed in Table S3. siRNAs were purchased from Sigma Aldrich or Santa Cruz Biotechnologies and transfected into cells using Lipofectamine 3000 (Thermo Fisher Scientific (Waltham, MA).

### In vitro generation of adipocytes and extraction of adipocyte exosomes

Primary adipocytes were isolated from bone marrow aspirates of mouse or human subjects as previously described [21]. Briefly, bone marrow aspirates were digested with 0.2% collagenase at 37 °C, centrifuged at 700 rpm for 10 min, and filtered through 200 µm membrane to separate from hematopoietic and stromal cells. The cells were further washed twice with 1 × PBS. In vitro generation and isolation of human mesenchymal stem cells (MSCs) and adipocytes [12, 22, 23] were performed as previously described. MSCs were maintained in mesenchymal stem cell medium (ScienCell Research Laboratory, Carlsbad, CA) and mature adipocytes were maintained in DMEM medium with 10% FBS. Mature adipocytes were characterized as previously described [12]. To collect adipocyte exosomes, we first cultured mature adipocytes alone or co-cultured with MM cells for 3 days. After removal of MM cells, adipocytes were cultured for another 6 days in DMEM medium with 10% exosome-free FBS. Exosomes were collected using total exosome isolation reagent (from cell culture media; Thermo Fisher Scientific) from filtered medium supernatants. Briefly, cell supernatant was mixed with isolation reagent at a 1-to-2 ratio, and the mixture was vortexed to form a homogenized solution. After overnight incubation at 4ºC, it was centrifuged at 10,000 g for 1 h at 4ºC. The pellet containing exosomes was resuspended in 1xPBS buffer. To characterize exosomes, they were examined by transmission electron microscopy (High Resolution Electron Microscopy Facility at MDACC) and by the expression of exosomal marker proteins such as HSP90, CD9, CD63, and CD81.

### Exosome uptake assay

Vybrant™ Dil cell-labeling solution (Dil), a red–orange fluorescent dye (Thermo Fisher Scientific), was used to label exosomes extracted from adipocyte supernatants. The Dil-labeled exosomes were added to the culture of ARP-1 cells. After 0, 12, and 24 h, ARP-1 cells were collected, fixed with 4% paraformaldehyde, stained with Alexa Fluor 488 Phalloidin for F-actin and DAPI, and then observed under confocal microscopy.

### Western blotting

Cells were harvested and lysed with 1 × lysis buffer (Cell Signaling Technology). To obtain exosomal proteins, exosomes were lysed in radioimmunoprecipitation assay (RIPA) lysis and extraction buffer and isolated using the total exosome RNA & Protein Isolation Kit (Thermo Fisher Scientific). Cell lysates were then subjected to SDS-PAGE, transferred to a polyvinylidene difluoride (PVDF) membrane, and immunoblotted with antibodies against HSP90, CD63, CD9, CD81, cytochrome c, c-Myc, IRF-4, c-MAF, cyclin-D1, p53, Rb, PTEN, EZH2, hnRNPA2B1, hnRNPU, Methyl-Lys, m^6^A, His-tag, METTL7A, and GAPDH (Cell Signaling Technology).

### Quantitative real-time PCR

Total RNA was isolated using a RNeasy kit (QIAGEN, Germany), while exosomal RNA were extracted from exosomes using total exosome RNA & Protein Isolation Kit. An aliquot of 1 μg of total RNA was subjected to reverse transcription (RT) with a SuperScript II RT-PCR kit (Invitrogen, Waltham, MA) according to the manufacturer instructions. Quantitative PCR was performed using SYBR Green Master Mix (Life Technologies) with the QuantStudio 6 Real-Time PCR System (Life Technologies). The primers used are listed in Table S4.

### Flow cytometry and ELISA

For Annexin V assay, apoptosis of treated cells (5 × 10^5^ cells/ sample) was detected by annexin V–APC/propidium iodide (PI) staining (Life Technologies). After 20 min of incubation at room temperature, cells were measured by a BD FACS Symphony A3 flow cytometer (BD Biosciences). Apoptotic cells were defined as the annexin V–positive cells. Cells from bone marrow were stained with anti-CD138 antibody or with TUNEL assay kit and measured by a BD FACS Symphony A3 flow cytometer (BD Biosciences). Results were analyzed using Flow Jo software. In addition, serum M-protein levels were measured using an ELISA kit (Thermo Fisher Scientific or Bethyl Laboratories, Montgomery, TX) according to the manufacturer instructions.

### Methyltransferase activity assay and dot blotting

The methyltransferase activity was measured using the Universal Methyltransferase Activity Assay kit (Abcam, Cambridge, United Kingdom) according to the manufacturer instructions. For each sample, 100 ng of RNAs and negative or positive control or different dilutions of METTL7A were mixed, and fluorescence signals were measured at 380ex/520em. For m^6^A dot blotting, 100 ng of RNAs were spotted onto nitrocellulose membrane using the Bio-Dot apparatus (Bio-Rad Laboratories, Hercules, CA). The membrane was UV-crosslinked, blocked with 5% nonfat dry milk, and then immunoblotted with anti-m^6^A antibody overnight at 4 °C. The membrane was washed and incubated with a secondary antibody. To ensure equal spotting of RNAs, the membrane was stained with 0.2% methylene blue in 0.4 M sodium acetate.

### Immunoprecipitation

Cells were lysed and incubated on ice for 15 min. The total protein lysate (500 µg/sample) was immunoprecipitated with an agarose-immobilized antibody at 4 °C overnight. After washing six times, the beads were spun down and resuspended in 30 μl of 1 × SDS buffer. After boiling for 5 min, pull-down samples were run on an SDS-PAGE gel along with a 5% input sample and transferred to a PVDF membrane for immunoblotting. IgG was used as a control and total cell lysates were used as input controls.

### RNA immunoprecipitation and RNA–protein pull down assays

RNA immunoprecipitation (RIP) was performed as previously described [24]. Cells were lysed in 1 × lysis buffer containing 100 mM of KCl, 5 mM of MgCl_2_, 10 mM of HEPES, 0.5% NP-40, and 1 mM of dithiothreitol, protease inhibitors, and RNase inhibitor (Thermo Fisher Scientific). After being spun down, the supernatants from cell lysates were incubated with a specific antibody or IgG control and then incubated with prewashed-protein G beads. After incubation, the mixtures were washed several times and the RNAs were isolated by phenol–chloroform-isoamyl alcohol and analyzed by quantitative PCR. For RNA–protein pull down assay, sense or antisense transcript of *LOC606724* was first biotinylated using RNA 3’-end desthiobiotynylation kit (Thermo Fisher Scientific) and then bound to the streptavidin magnetic beads. Cell lysates pulled down by the magnetized biotinylated-labeled sense or antisense transcript of *LOC606724* were either sent for mass spectrometric analysis (The Proteomics Facility at MDACC) or immunoblotted against anti-hnRNPA2B1 or anti-hnRNPU antibodies. Inputs were used as control.

### RNA immunoprecipitation sequencing (MeRIP-seq)

Total RNA was extracted using Trizol reagent (Life Technologies) following the manufacturer's procedure. MeRIP-seq were conducted by LC Sciences, Houston, TX. Briefly, poly(A) mRNA was isolated and purified from 50 μg of total RNA with poly-T oligo attached magnetic beads (Life Technologies), which were then fragmented into ~ 100-nt-long oligonucleotides. The cleaved RNA fragments were first incubated with an m^6^A-specific antibody for 2 h at 4℃ and then with protein-A beads, and finally eluted with elution buffer. Eluted m^6^A-containing fragments and untreated input control fragments are converted to final cDNA library in accordance with a strand-specific library preparation by dUTP method. The average insert size for the paired-end libraries was ~ 100 ± 50 bp. Paired-end 2 × 150 bp sequencing was performed on an Illumina Novaseq™6000 platform.

### In vivo mouse experiments

NOD-*scid* IL2Rg^null^ (NSG) and C57BL/6 mice were purchased from The Jackson Laboratory, Bar Harbor, ME. Inducible adipose-specific *Ezh2*^flox/flox^ knockout mice were established as previously described with mice purchased from The Jackson Laboratory [12]. All mice were maintained in American Association of Laboratory Animal Science-accredited facilities and all in vivo mouse studies were approved by the Institutional Animal Care and Use Committees of HMRI.

In the xenografted-MM mouse model, ARP-1 cells (5 × 10^5^ cells/mouse) were intrafemorally injected into the NSG mice. After 2 weeks, mice were intraperitoneally injected with bortezomib (0.5 mg/kg) alone or in combination with tazemetostat (0.5 g/kg) via oral gavage three times a week for 3 weeks. Mice receiving equal amounts of vehicle served as the control. Tumor burden was evaluated by serum M-protein level and strength of bioluminescent signal.

The primers used for genotyping of inducible adipose-specific *Ezh2*^flox/flox^ knockout mice were described previously [12]. Mice (*Adipoq*-*CreER*-*Ezh2*^flox/flox^) were given 75 mg/kg tamoxifen or corn oil intraperitoneally daily for 5 consecutive days to generate mice with deletion of the *Ezh2* gene in adipocytes or control mice *(40)*. In addition, western blotting was used to confirm the absence of EZH2 expression in the bone marrow of randomly selected mice. To establish MM in those mice, Vk*MYC cells (1 × 10^6^ cells/mouse) were injected intrafemorally into wild type and *Ezh2*-knockout mice. After 2 weeks, mice were treated intraperitoneally with bortezomib (0.5 mg/kg) three times a week for 4 weeks. Mice receiving equal amounts of vehicle served as the control. Tumor burden was evaluated by serum M-protein levels.

In both models, bone marrow was extracted and aliquots of the cells were used to determine the ratios of CD138^+^ MM cells or percentage of TUNEL^+^ MM cells by flow cytometry. CD138^+^ MM cells were isolated from bone marrow aspirates and evaluated for mRNA expression.

### Statistics

Statistical significance was analyzed using the GraphPad Prism (San Diego, CA) with two tailed unpaired Student *t*-tests for comparison of two groups and one-way ANOVA for comparison of more than two groups. Pearson’s correlation analysis was used to examine the correlation between two variables and Kaplan–Meier analysis was used in survival analysis. *P* values less than 0.05 were considered statistically significant. Data shown as mean ± SD, representative of three independent experiments.

## Results

### Adipocyte-derived exosomes protect MM cells against chemotherapy-induced apoptosis

**Fig. 1:**
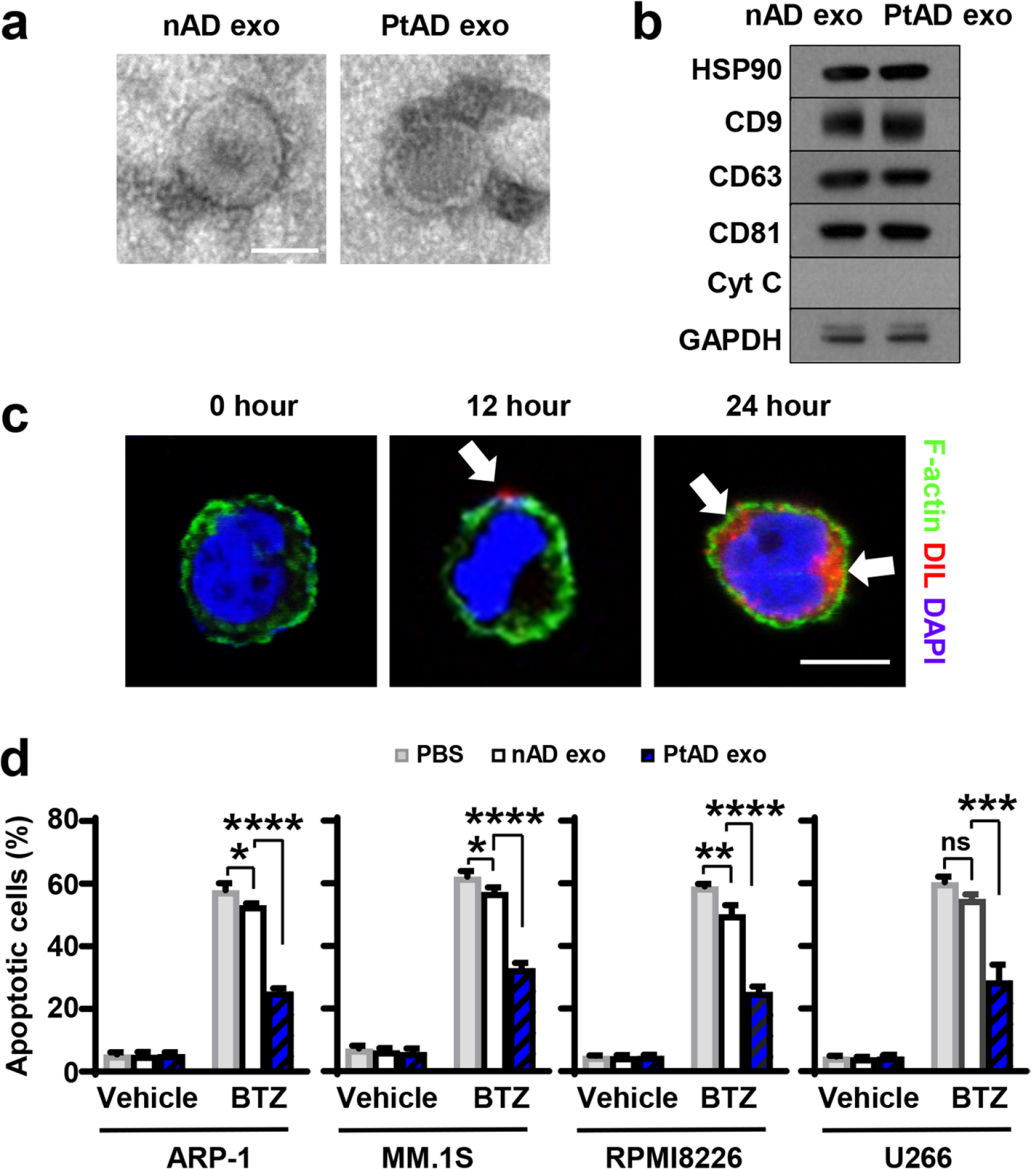
Exosomes derived from patients’ adipocytes protect MM cells against chemotherapeutic treatment. **a-c**. Characterization of adipocyte-derived exosomes. **a.** Representative images of exosomes derived from normal adipocytes (nADs) and MM patients’ adipocytes (PtADs) under a transmission electron microscope. Scale bar, 50 nm. **b.** Representative western blots show the levels of exosome markers HSP90, CD9, CD63, and CD81 in exosomes from nADs and PtADs. Levels of cytochrome c (Cyt c) served as negative control. **c.** Confocal microscopy shows the representative images of ARP-1 cells that were exposed to Dil pre-stained adipocyte-derived exosomes. ARP-1 cells were then stained with Alexa Fluor 488 Phalloidin for F-actin and DAPI for nucleus staining. Scale bar, 10 μm. **d.** Annexin V-binding assay shows the percentages of apoptotic MM cells treated with 5 nM bortezomib (BTZ) and without or with nAD and PtAD exosomes after 24 h. Data shown as mean ± SD. ns, non-significant; *P < 0.05; **P < 0.01; ***P < 0.001; ****P < 0.0001. *P* values were determined by one-way ANOVA

To explore the potential role of adipocyte-derived exosomes in MM, we first cultured adipocytes isolated from normal bone marrow (nADs) or the marrow of patients with newly diagnosed MM (PtADs) for 2 days. We then examined exosomes collected from the culture medium of adipocytes using transmission electron microscopy. In line with previous reports [25], we observed that adipocyte-derived exosomes exhibited a characteristic cup-shape morphology with a diameter between 30–150 nm (Fig. 1a). This was further confirmed by the expression of exosome markers (Fig. 1b). The nAD-derived and PtAD-derived exosomes shared similar properties such as morphology, size, and the expression level of exosomal markers (Fig. 1a, b). To investigate whether exosomes can be taken up by MM cells, we incubated the human MM cell line ARP-1 cells with nAD- or PtAD-derived exosomes labeled with the fluorescent dye DiL that brightens as it incorporates into the membrane. Confocal microscopy showed that DiL signals were detected on the ARP-1 cell membrane surface after 12 h and moved inside cytoplasm 24 h after exposure to adipocyte exosomes (Fig. 1c and Fig. S1). This observation suggests that adipocyte-derived exosomes have the capability to internalize into MM cells.

To determine the functional role of adipocyte-derived exosomes in MM therapeutic response, we added nAD- or PtAD-derived exosomes and the chemotherapy drug bortezomib or PBS vehicle control to the cultures of ARP-1, MM.1S, RPMI8226, and U266 MM cells for 24 h. While the bortezomib treatment induced apoptosis in MM cells, addition of nAD exosomes slightly reduced the efficacy of bortezomib (Fig. 1d). Moreover, when we cultured MM cells with exosomes collected from PtADs, the protective effect became more evident, as more MM cells survived bortezomib treatment than those cultured with exosomes collected from nADs (Fig. 1d), suggesting that the effect by adipocyte exosomes may potentially contribute to MM therapeutic response.

### MMAD-secreted exosomes have more effects to induce MM drug resistance

**Fig. 2:**
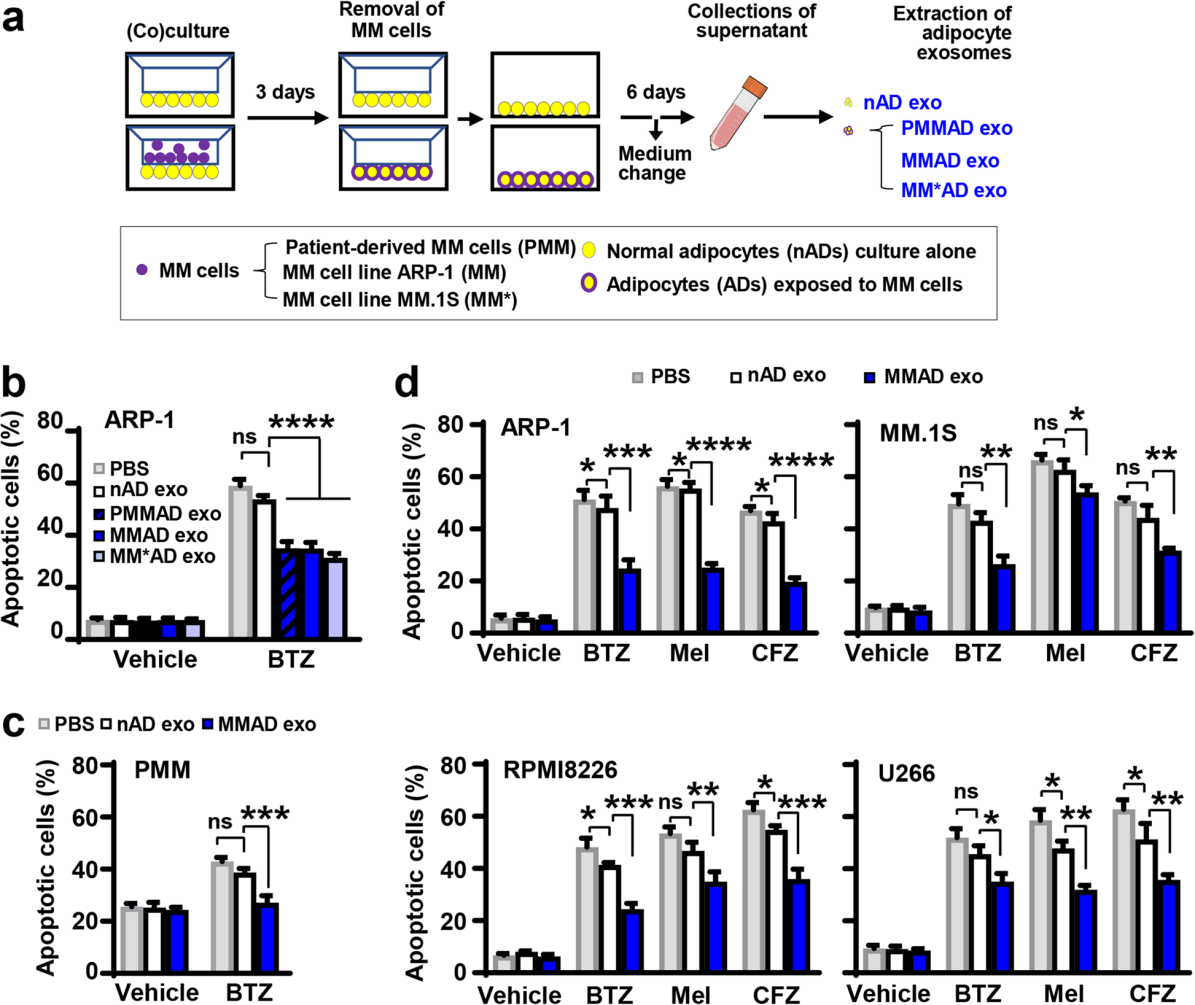
MM cells exposed to exosomes derived from MM-associated adipocytes are more resistant to chemotherapeutic drugs than those derived from normal adipocytes. **a**. Schematic for co-cultures of MM cells and adipocytes, and collection of the exosomes from adipocytes. **b-d.** MM cells were treated with 5 nM bortezomib (BTZ), or 25 μM melphalan (Mel), or 25 nM carfilzomib (CFZ) and with or without adipocyte exosomes for 24 h. Addition of vehicle or PBS served as control for drugs or exosomes, respectively. **b.** Annexin V-binding assay shows the percentages of apoptotic ARP-1 cells treated with bortezomib and exosomes derived from nADs, PMMADs, and MMADs. **c.** Annexin V-binding assay shows the percentages of apoptotic primary MM cells, isolated from bone marrow aspirates of patients with newly diagnosed MM, treated with bortezomib and exosomes derived from nADs or MMADs. **d.** Annexin V-binding assay shows the percentages of apoptotic MM cells treated with various chemotherapeutic drugs, vehicle control, and exosomes derived from nADs or MMADs. Data shown as mean ± SD. ns, non-significant; *P < 0.05; **P < 0.01; ***P < 0.001; ****P < 0.0001. *P* values were determined by one-way ANOVA

Since exosomes collected from adipocytes of MM patients exhibited more protective effects, we considered if MM cells might influence adipocyte exosome-mediated drug resistance. To examine our hypothesis, we first used a co-culture system to mimic the interaction between MM cells and adipocytes. On the opposite side of the transwell, nADs and MM cells, either from cell lines (ARP-1 and MM.1S) or primary malignant plasma cells isolated from bone marrow aspirates of newly diagnosed MM patients, were seeded and incubated for 72 h (Fig. 2a). To differentiate it from controls, which are nADs cultured alone, the adipocytes exposed to MM cells were designated as MM-associated adipocytes (MMADs) (Fig. 2a). Exosomes collected from the culture medium of nADs or MMADs were added to cultures of ARP-1 MM cells treated with bortezomib. Compared to those from nADs, the exosomes secreted from MMADs had more inhibitory effects on bortezomib-induced apoptosis in ARP-1 cells (Fig. 2b). Culturing with MMAD exosomes also reduced the efficacy of bortezomib treatment in primary malignant plasma cells isolated from patient bone marrow aspirates (Fig. 2c). We observed a similar trend in multiple MM cell lines (ARP-1, MM.1S, RPMI8226, and U266) treated with different chemotherapeutic drugs such as bortezomib, melphalan, and carfilzomib (Fig. 2d and Fig. S2). These results indicate that MM cells may enhance the effect of adipocyte exosomes on the resistance of MM cells to chemotherapy.

### Adipocyte exosome-carried LncRNAs inhibit MM cell apoptosis

**Fig. 3:**
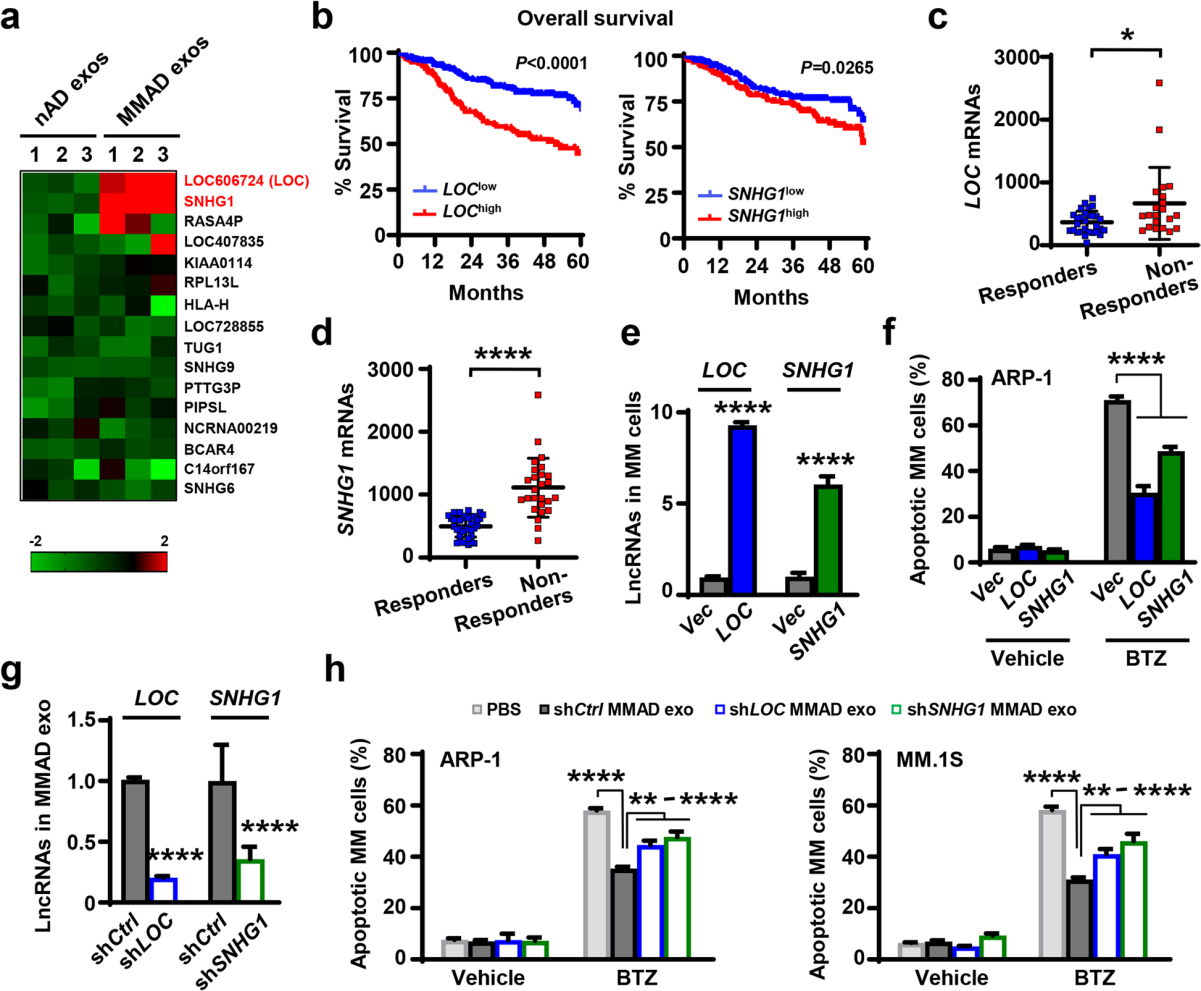
Adipocyte exosomal LncRNAs contributes to MM drug resistance. **a.** Quantitative real-time PCR analysis shows the relative expression of a panel of LncRNAs in MM cells exposed to exosomes derived from nAD or MMADs. **b.** Kaplan–Meier analysis shows the overall survival of MM patients with low or high expression of *LOC606724* (*LOC,* n = 400) or *SNHG1* (n = 400) in MM cells analyzed from MMRF’s CoMMpass database. **c & d.** The expression of *LOC* (**c**, n = 50) or *SNHG1* (**d**, n = 63) in MM patients who responded (Responders) or did not respond (Non-Responders) to bortezomib-based treatment from CoMMpass database. **e & f.** ARP-1 cells were overexpressed with *LOC* or *SNHG1*. Cells transfected with empty vector (*Vec*) served as control. **e.** Quantitative real-time PCR analysis shows the relative expression of *LOC* and *SNHG1* in ARP-1 cells overexpressed with respective genes. **f.** Annexin V-binding assay shows the percentages of apoptotic ARP-1 cells overexpressed with *LOC* or *SNHG1* treated with vehicle or bortezomib (BTZ, 5 nM) for 24 h. **g & h.** MMADs were infected with lentiviral particles carrying shRNAs targeting *LOC* or *SNHG1*. **g.** Quantitative real-time PCR analysis shows the relative level of exosomal *LOC* or *SNHG1* collected from sh*LOC* or sh*SNHG1* MMADs. **h.** Annexin V-binding assay shows the percentages of apoptotic MM cells treated with vehicle or 5 nM bortezomib for 24 h with sh*LOC* or sh*SNHG1* MMAD-derived exosomes. MMADs treated with vehicle or non-target shRNA (sh*Ctrl*) served as control. Data shown as mean ± SD. *P < 0.05; **P < 0.01; ****P < 0.0001. *P* values were determined by Student *t*-tests for comparison of two groups and one-way ANOVA for comparison of more than two groups

Since exosomes contain a rich collection of LncRNAs and LncRNAs have been implicated in tumor drug resistance [18, 26, 27], we considered if LncRNAs from adipocyte exosomes influence MM cell response to chemotherapy. Previous studies have identified an array of LncRNAs overexpressed in MM patients compared to normal subjects (Gene Expression Omnibus GSE5900 and GSE 2658, [28]). We examined these LncRNAs in ARP-1 MM cells with the addition of exosomes extracted from nAD or MMAD. We found that LncRNAs such as *LOC606724* and *SNHG1* were upregulated in the presence of MMAD exosomes compared to those with exosomes from nADs (Fig. 3a), suggesting that MM cells may be able to uptake LncRNAs exogenously from extracellular components such as adipocyte exosomes. To determine the clinical relevance of those LncRNAs in MM, we analyzed the data from MMRF CoMMpass RNA-seq database. We found a strong positive correlation between the expression levels of *LOC606724* and *SNHG1* in MM cells and patient overall survival (Fig. 3b). In the cohort of patients treated with bortezomib-based therapies, the levels of *LOC606724* and *SNHG1* mRNAs in MM cells were much higher in non-responders than those who responded to the treatment (Fig. 3c, d). These results strongly suggest the potential association between LncRNAs from adipocyte exosomes and MM drug resistance.

To examine the effects of *LOC606724* or *SNHG1* on MM cell response to bortezomib treatment, we overexpressed them in ARP-1 MM cells (Fig. 3e). We found that the overexpression significantly reduced the efficacy of bortezomib on ARP-1 cell apoptosis (Fig. 3f). To determine their impacts on adipocyte exosome-mediated MM drug resistance, we used specific shRNAs targeting *LOC606724* or *SNHG1* to knock down their expression in MMADs and found reduced levels of exosomal *LOC606724* or *SNHG1* (Fig. 3g). The adipocytes expressing non-targeted shRNAs served as control (Fig. 3g). We then extracted exosomes from sh*Ctrl*, sh*LOC606724*, or sh*SNHG1* MMADs, added them to the cultures of MM cells and found that the knockdown significantly enhanced the efficacy of bortezomib treatment in ARP-1 or MM.1S MM cells (Fig. 3h). These results suggest that MMAD exosome-carried LncRNAs can protect MM cells against chemotherapy-induced apoptosis.

**Fig. 4:**
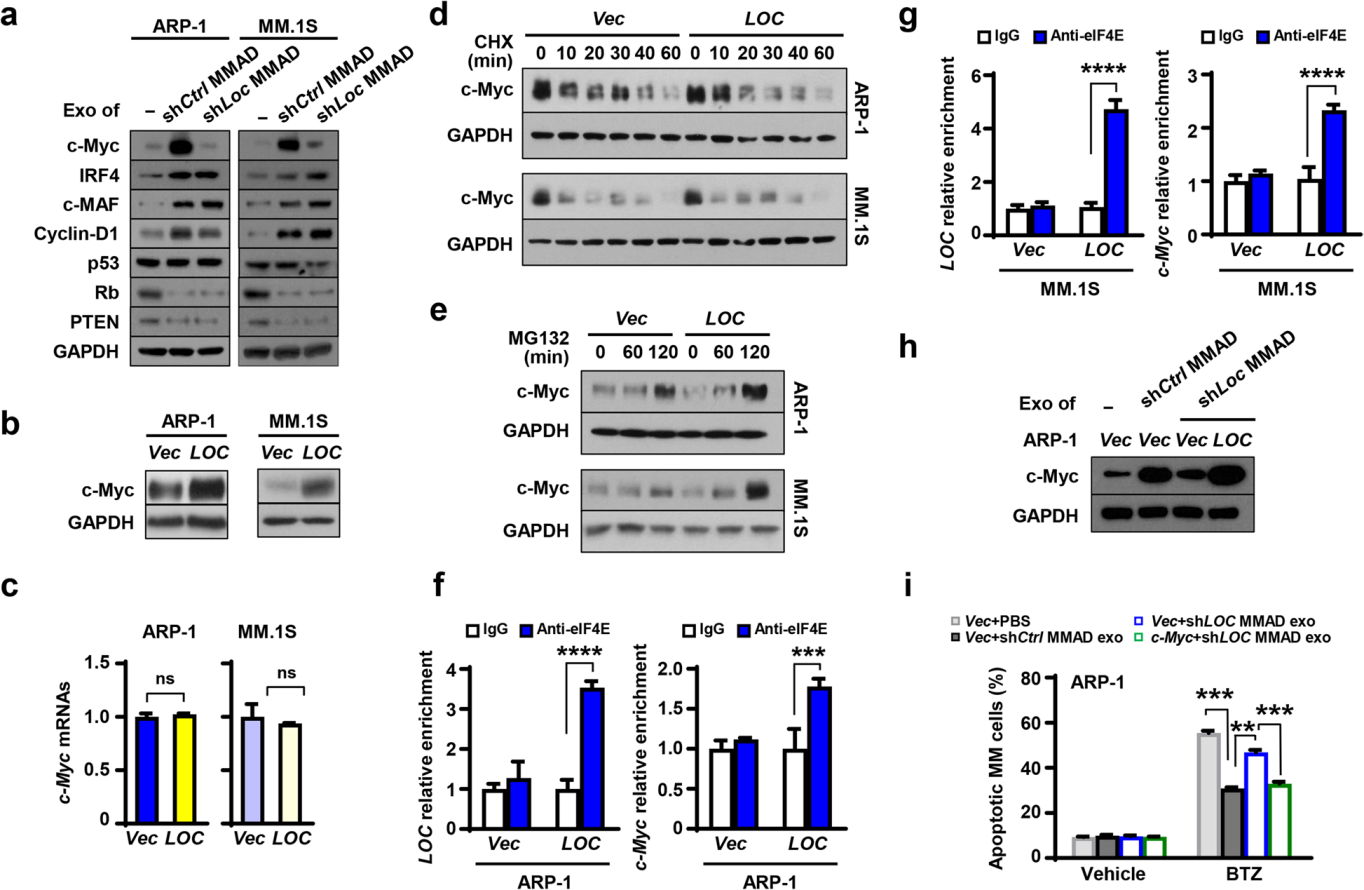
*LOC606724* enhances c-Myc protein synthesis in MM cells. **a.** Western blotting shows the levels of oncogenic or tumor suppressive proteins in ARP-1 and MM.1S cells cultured with shLOC MMAD-derived exosomes. Treatment of PBS or exosomes from sh*Ctrl* MMADs served as control. **b & c**. The levels of c-Myc proteins (**b**) and mRNAs (**c**) in ARP-1 or MM.1S cells overexpressed with empty vector (*Vec*) or *LOC606724 (LOC)*. **d.** Western blots show the levels of c-Myc protein in ARP-1 or MM.1S cells overexpressed with *Vec* or *LOC* treated with 100 µM cycloheximide (CHX) for various length of time. **e.** Western blots show the levels of c-Myc protein in ARP-1 or MM.1S cells overexpressed with *Vec* or *LOC* treated with 10 µM MG132 for 0, 60, and 120 min. **f & g.** Cell lysates of MM cell ARP-1 (**f**) or MM.1S (**g**) overexpressed with *Vec* or *LOC* were pulled down by an anti-Eif4E antibody, and RIP assay shows the relative enrichment of *LOC* and *c-Myc* in those cells. Cell lysates pulled by IgG served as control. **h** & **i**. ARP-1 cells overexpressing empty vector or c-Myc were treated with 5 nM bortezomib in the presence of vehicle or exosomes derived from shCtrl or shLOC606724 MMADs for 24 h. Western blotting shows the levels of c-Myc (**h**) and Annexin V-binding assay shows the percentages of apoptotic ARP-1 cells (**i**). For western blotting, GAPDH served as loading control. Data shown as mean ± SD. ns, non-significant; **P < 0.01; ***P < 0.001; ****P < 0.0001. *P* values were determined by Student *t*-tests for comparison of two groups and one-way ANOVA for comparison of more than two groups

While *SNHG1* has been shown to regulate tumor growth, the biological function of *LOC606724* in tumors is unknown. We therefore investigated how *LOC606724* protects MM cells from apoptosis. We incubated MM cells with exosomes collected from sh*Ctrl* or sh*LOC606724* MMADs and examined the expression of several oncogenes and tumor suppressors whose levels are known to be changed in MM. We found that the expressions of some were altered in MM cells exposed to exosomes collected from sh*Ctrl* MMADs compared to those in MM cells cultured alone (Fig. 4a). However, the effect of the *LOC606724* knockdown in adipocytes was most obvious on the expression of oncogenetic protein c-Myc, whose MMAD exosome-induced upregulation was abrogated (Fig. 4a). Moreover, we observed enhanced c-Myc protein levels without affecting *c-Myc* mRNAs in ARP-1 or MM.1S MM cells overexpressed with *LOC606724* (Fig. 4b and c). These results indicate that *LOC606724* upregulates MM cell c-Myc protein at the post transcriptional level.

To examine whether *LOC606724* could regulate the synthesis or degradation of c-Myc protein, we added the respective inhibitor to the cultures of ARP-1 or MM.1S MM cells. We found that inhibition of protein synthesis by cycloheximide reduced c-Myc protein levels in a similar pattern between the vector control and *LOC606724*-overexpressing MM cells (Fig. 4d), suggesting that the rate of protein degradation remains the same and may not be affected by *LOC606724*. While inhibition of protein degradation by MG132 enhanced the accumulation of c-Myc protein in MM cells, we observed more accumulation in *LOC606724*-overexpressing MM cells than in control cells (Fig. 4e), indicating that *LOC606724* modulates c-Myc proteins in MM cells at the translational level. Since eIF4E is a key molecule in the process of protein translation, we considered if *LOC606724* could bind to eIF4E protein. We performed RIP assay by pulling down the lysates of MM cells using an antibody against eIF4E, and observed higher enrichment of *LOC606724* and *c-Myc* in the immunoprecipitants of *LOC606724*-overexpressing MM cells than in control cells (Fig. 4f and g). We then performed a rescue experiment by overexpressing empty vector or *c-Myc* in MM cells (Fig. 4h). While addition of exosomes extracted from MMAD with knock-down *LOC606724* sensitized the therapeutic effect of bortezomib, overexpression of c-Myc reversed such effect (Fig. 4i)*.* These results illustrate the interactions among *LOC606724*, eIF4E, and *c-Myc*, suggesting that *LOC606724* functions as a bridge to link eIF4E and c-Myc, leading to eIF4E-mediated c-Myc protein synthesis. Together, our results suggest that adipocyte exosomal LncRNAs may play an important role in MM therapeutic responses.

### MM cells enhance LncRNA enrichment into adipocyte exosomes

**Fig. 5:**
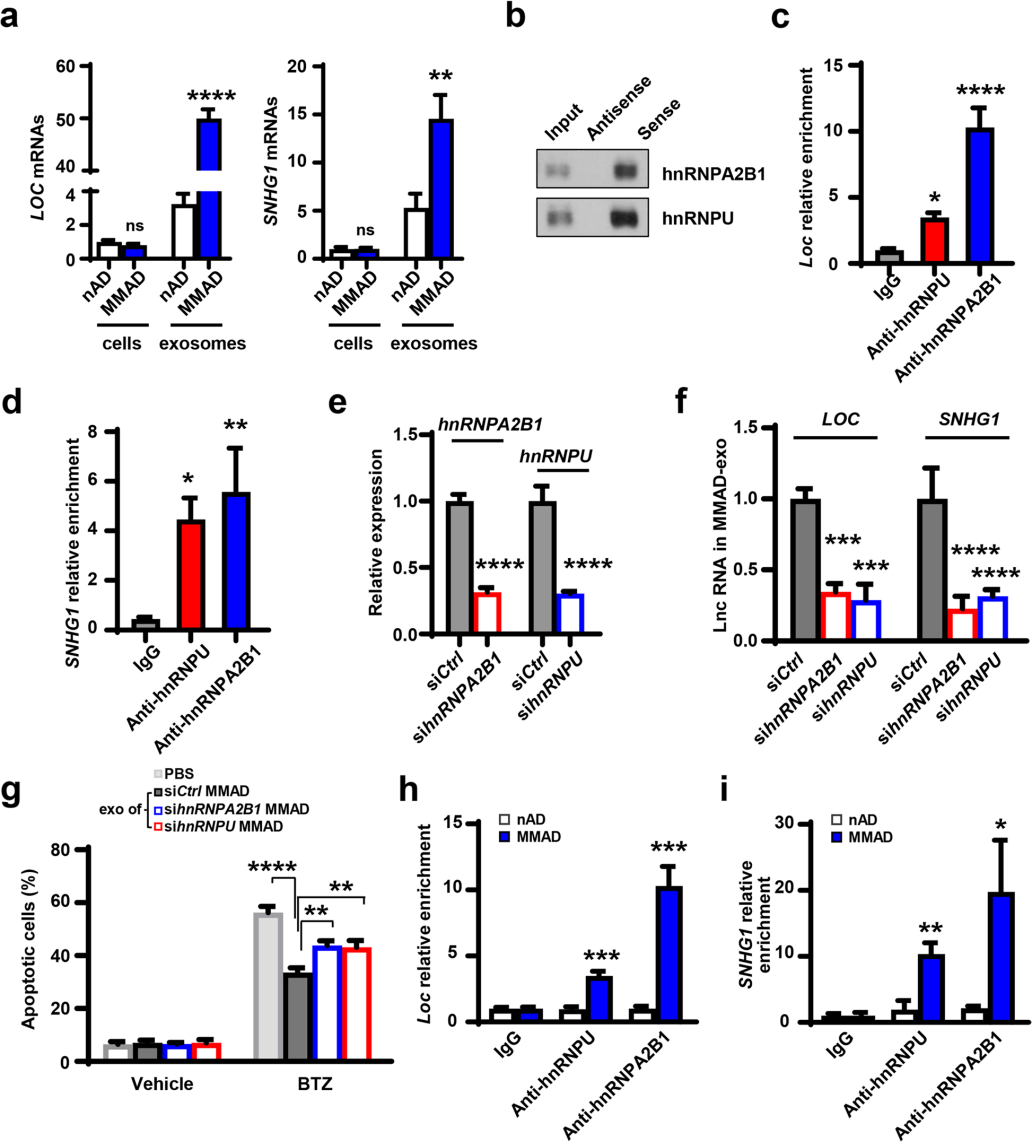
Interaction with RNA binding proteins induces LncRNA packaging into adipocyte exosomes. **a.** Quantitative real-time PCR analysis shows the relative level of cellular and exosomal *LOC606724* (*LOC)* and *SNHG1* in nADs or MMADs. **b.** Western blotting shows the level of hnRNPA2B1 or hnRNPU in the lysate of MMADs using RNA pull down by biotin-labeled sense or antisense transcript of *LOC*. Inputs served as control. **c & d.** RIP assay shows the relative enrichment of *LOC* or *SNHG1* in the immunoprecipitates of MMAD lysates pulled down by anti-hnRNPA2B1 (**c**) or anti-nRNPU (**d**) antibodies. **e.** RNA interference efficacy of si*hnRNPA2B1* and si*hnRNPU* in MMADs. **f.** Quantitative real-time PCR analysis shows the relative level of exosomal *LOC* and *SNHG1* derived from MMADs carrying si*hnRNPA2B1* or si*hnRNPU*. **g.** Annexin V-binding assay shows the percentages of apoptotic ARP-1 cells treated with 5 nM bortezomib (BTZ) and exosomes derived from MMADs carrying si*hnRNPA2B1* or si*hnRNPU*. ARP-1 cells treated with vehicle or exosomes derived from MMADs carrying si*Ctrl* served as controls. **h & i.** RIP assay shows the relative enrichment of *LOC* or *SNHG1* in the immunoprecipitates from lysates of nADs or MMADs pulled down by anti-hnRNPA2B1 (**h**) or anti-hnRNPU (**i**) antibodies. Cell lysates pulled by IgG served as control. Data shown as mean ± SD. ns, non-significant; *P < 0.05; **P < 0.01; ***P < 0.001; ****P < 0.0001. *P* values were determined by Student *t*-tests for comparison of two groups and one-way ANOVA for comparison of more than two groups

We next investigated whether MM cells could regulate LncRNAs in adipocytes. As shown in Fig. 5a, there was little difference on the cellular levels of LncRNAs between nADs and MMADs, but the exosomal levels were remarkably higher in MMADs compared to those in nADs. We therefore considered if MM cells could enhance LncRNA enrichment into adipocyte exosomes. Using RNA pull down assay, we first examined the RNA binding proteins such as hnRNPA2B1 and hnRNPU, required for the packaging of non-coding RNAs into exosomes [26, 29]. We were able to detect the presence of hnRNPA2B1 and hnRNPU in the pulldown of MMAD cell lysates with a biotin-labeled sense transcript of *LOC606724* (Fig. 5b). The antisense transcript of *LOC606724* served as the negative control. Using antibodies against hnRNPA2B1 or hnRNPU, RIP assay showed the enrichment of *LOC606724* or *SNHG1* in adipocytes (Fig. 5c and d). These results suggest the interaction between LncRNAs and RNA binding proteins. Next, we knocked down the expression of *hnRNPA2B1* or *hnRNPU* in MMADs using specific siRNAs (Fig. 5e) and found that the knockdown significantly reduced levels of *LOC606724* and *SNHG1* in MMAD exosomes (Fig. 5f). When those MMAD exosomes were added into cultures of MM cells, the efficacy of bortezomib improved significantly by inducing more MM cells into apoptosis (Fig. 5g). Not surprisingly, we observed much higher enrichment of *LOC606724* or *SNHG1* in the immunoprecipitates of MMADs pulled down by the anti-hnRNPA2B1 or anti-hnRNPU antibody when compared to the immunoprecipitates of nAD pull downs (Fig. 5h and i). These results indicate that through binding to RNA binding proteins, more adipocyte LncRNAs are packaged into exosomes when exposed to MM cells.

### Identification of METTL7A-mediated m^6^A methylation of LncRNAs in adipocytes

**Fig. 6:**
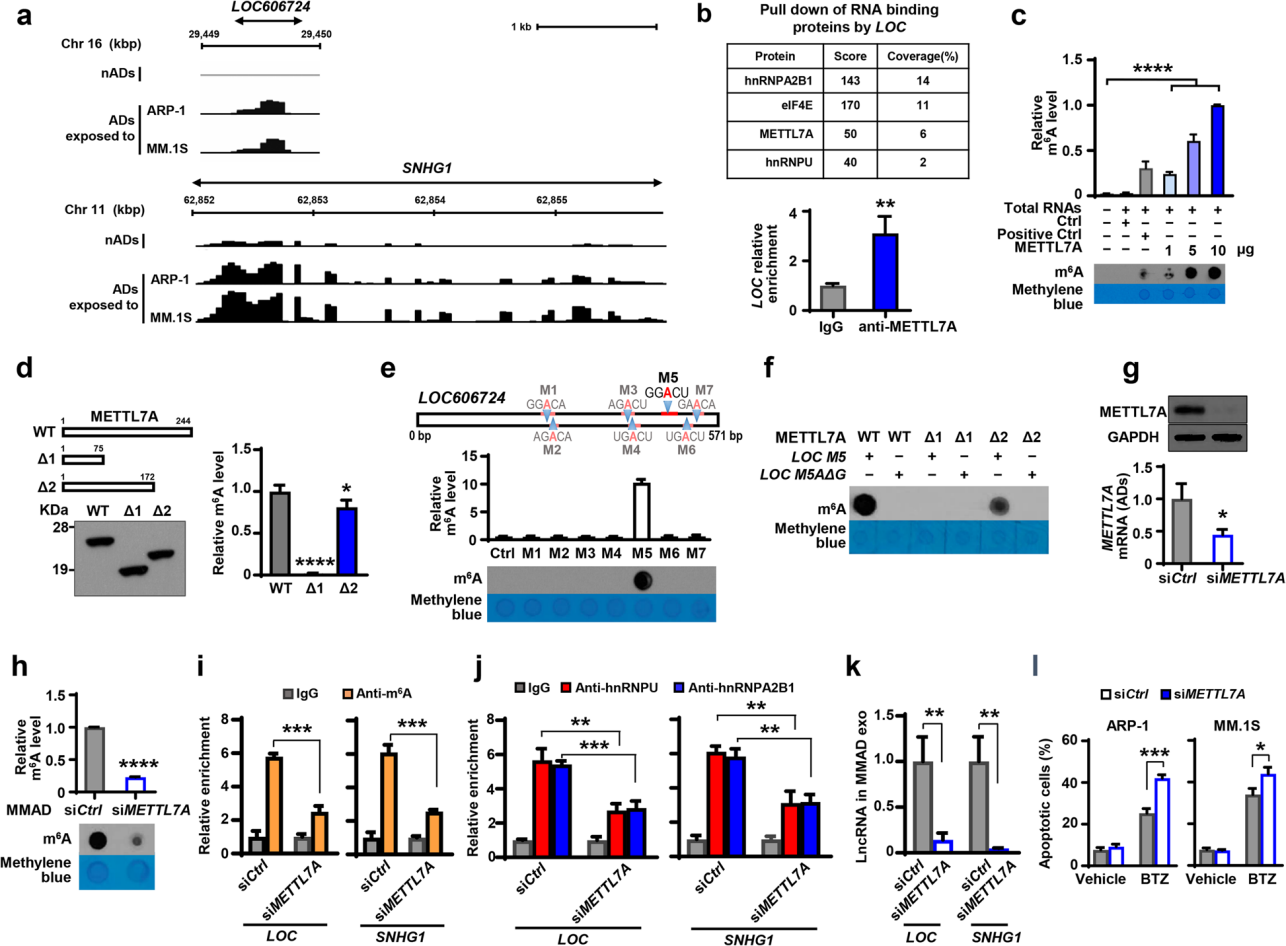
METTL7A mediates RNA m^6^A methylation in LncRNAs. **a.** Transcriptome-wide mapping of MeRIP-seq shows the methylation profile of *LOC606724* or *SNHG1* in nADs or nADs exposed to MM ARP-1 or MM.1S cells. **b.** Mass spectrometry identifies the proteins in MMADs that were pulled down by biotin-labeled sense transcript of *LOC606724 (LOC)*. RIP assay using anti-METTL7A antibodies shows the relative enrichment of *LOC* in the immunoprecipitates from MMAD lysates. Cell lysates pulled down by IgG served as control. **c-f.** Full length or truncated forms of His-tagged METTL7A were expressed and purified. **c.** Methyltransferase activity assay and dot blotting show the relative m^6^A levels in the mixture of various amount of His-tagged METTL7A and RNA substrates. **d.** Western blotting shows the full length (WT) and two truncated forms (△1 and △2) METTL7A protein immunoblotted with anti-His antibody and methyltransferase activity assays shows their relative m^6^A levels. **e.** Schematic illustration of seven putative METTL7A binding motifs and their respective custom RNA oligonucleotides (red or light red bar) on *LOC* transcript. Methyltransferase activity assay and dot blotting show relative m^6^A levels in the mixture of custom RNA oligonucleotides and full length METTL7A. **f.** Dot blotting shows relative m^6^A levels in the mixture of different combination of RNA oligonucleotides containing wild-type M5 motif (*LOC M5*) or mutated M5 motif (GGGCU, *LOC M5AΔG*) with full length or truncated METTL7A. **g-l.** MMADs were transfected with siRNA against METTL7A (si*METTL7A*). Cells transfected with scrambled siRNA served as control (si*Ctrl*). **g.** The levels of METTL7A proteins and mRNAs in si*Ctrl* or si*METTL7A* MMADs. **h.** Methyltransferase activity assay and dot blotting show relative m^6^A levels in si*Ctrl* or si*METTL7A* MMADs. **i & j.** RIP assay shows the relative enrichment of *LOC* or *SNHG1* in the immunoprecipitates from lysates of si*Ctrl* or si*METTL7A* MMADs pulled down by anti-m^6^A antibodies (**i**) or by anti-hnRNPU or anti-hnRNPA2B1 antibodies (**j**). **k.** Quantitative real-time PCR analysis shows the relative level of exosomal *LOC* and *SNHG1* derived from si*Ctrl* or si*METTL7A* MMADs. **l.** Annexin V-binding assay shows the percentages of apoptotic MM cells treated with 5 nM of bortezomib (BTZ) and exosomes derived from si*Ctrl* or si*METTL7A* MMADs. Treatment with vehicle served as control. In western blotting, GAPDH served as loading control. *P < 0.05; **P < 0.01; ***P < 0.001; ****P < 0.0001. *P* values were determined by Student *t*-tests for comparison of two groups and one-way ANOVA for comparison of more than two groups

Methylation is a key mechanism for RNA modification involved in cancer development. The most common RNA modification is the methylation at N6 position in adenosine (m^6^A) [30]. We thus screened adipocytes using methylated RNA immunoprecipitation sequencing (MeRIP-seq). As shown in Fig. 6a, the methylation levels of *LOC606724* and *SNHG1*, for example, were much higher in MMADs than those in nADs, indicating the methylation of LncRNAs in MMADs. By pulling down MMAD cellular lysates with biotin-labeled sense or antisense transcript of *LOC606724*, mass spectrometric analysis confirmed the presence of two RNA binding proteins, hnRNPA2B1 and hnRNPU, in the sense transcript (Fig. 6b, top). In addition, we observed the presence of methyltransferase like 7A (METTL7A) protein in the pulldown (Fig. 6b, top). RIP assay further showed the enrichment of *LOC606724* in the immunoprecipitates pulled down by the anti-METTL7A antibody (Fig. 6b, bottom). These results indicate the interaction between LncRNAs and METTL7A protein.

METTL7A belongs to the METTL family [31, 32]. Although several members of this family have been shown to have m^6^A RNA methyltransferase activity [33–35], it is not clear whether METTL7A has such ability. We constructed and purified the recombinant His-tagged METTL7A fusion protein. In vitro methyltransferase activity assay showed that incubation of substrates with the His-tagged METTL7A protein significantly increased m^6^A levels in a dose-dependent manner (Fig. 6c). To pinpoint the region(s) on METTL7A protein that may possess the enzyme activity, we constructed two truncated forms of METTL7A proteins: △1 (1–75 aa) and △2 (1–172 aa). While the incubation of substrates with full length or △2 of METTL7A protein significantly increased m^6^A levels, incubation with △1 displayed little activity (Fig. 6d). These results indicate that the m^6^A RNA methyltransferase activity mostly comes from the region in 76–172 aa of METTL7A protein.

Next, we examined the potential site(s) on LncRNAs that could be methylated by METTL7A. Sequence-based RNA adenosine methylation site predictor (SRAMP) software identified seven potential methylation motifs (M1 to M7) on *LOC606724*. Using custom RNA oligonucleotide that was specific for each candidate motif, we conducted in vitro methylation assays. We found that m^6^A levels were significantly increased when METTL7A protein was incubated with RNA oligonucleotides covering the M5 motif, which contains an adenosine at 481 on *LOC606724,* while the incubation with negative control or any of the other motifs yielded no such effect (Fig. 6e). Furthermore, we mutated adenosine to guanosine at 481 bp of the M5 oligonucleotide (M5A△G), and m^6^A activities were attenuated with either full length or truncated METTL7A protein (Fig. 6f). Likewise, incubation of wild-type M5 oligonucleotide with full length or truncated △2, but not △1, of METTL7A protein increased m^6^A levels (Fig. 6f). These results indicate that the sequence between 76 to 172 aa of METTL7A contributes to the methylation of adenosine at 481 of *LOC606724*.

To examine the impact of METTL7A-mediated RNA methylation on LncRNA enrichment, we knocked down the expression of METTL7A in MMADs using the specific siRNA (si*METTL7A*, Fig. 6g). Non-targeted siRNA served as control (si*Ctrl*). We found that knockdown of METTL7A significantly reduced m^6^A levels in MMADs (Fig. 6h). The m^6^A‐RNA immunoprecipitation assay showed lower m^6^A levels on *LOC606724* and *SNHG1* in si*METTL7A* MMADs than those in si*Ctrl* cells (Fig. 6i). Furthermore, we found the reduced enrichment of *LOC606724* or *SNHG1* to RNA binding proteins hnRNPA2B1 or hnRNPU (Fig. 6j), as well as the drastic reductions on the level of exosomal *LOC606724* or *SNHG1* in si*METTL7A* MMADs compared to those in si*Ctrl* MMADs (Fig. 6k). Functionally, incubation with the exosomes of si*METTL7A* MMADs improved the efficacy of bortezomib treatment as more MM cells died comparing to those incubated with si*Ctrl* MMAD exosomes (Fig. 6l). Together, these results indicate that METTL7A with a methyltransferase activity contributes to m^6^A methylation on LncRNAs, and the methylation of LncRNA allows enrichment into adipocyte exosomes.

### MM cells enhance METTL7A activity in adipocytes through EZH2-mediated protein methylation

**Fig. 7:**
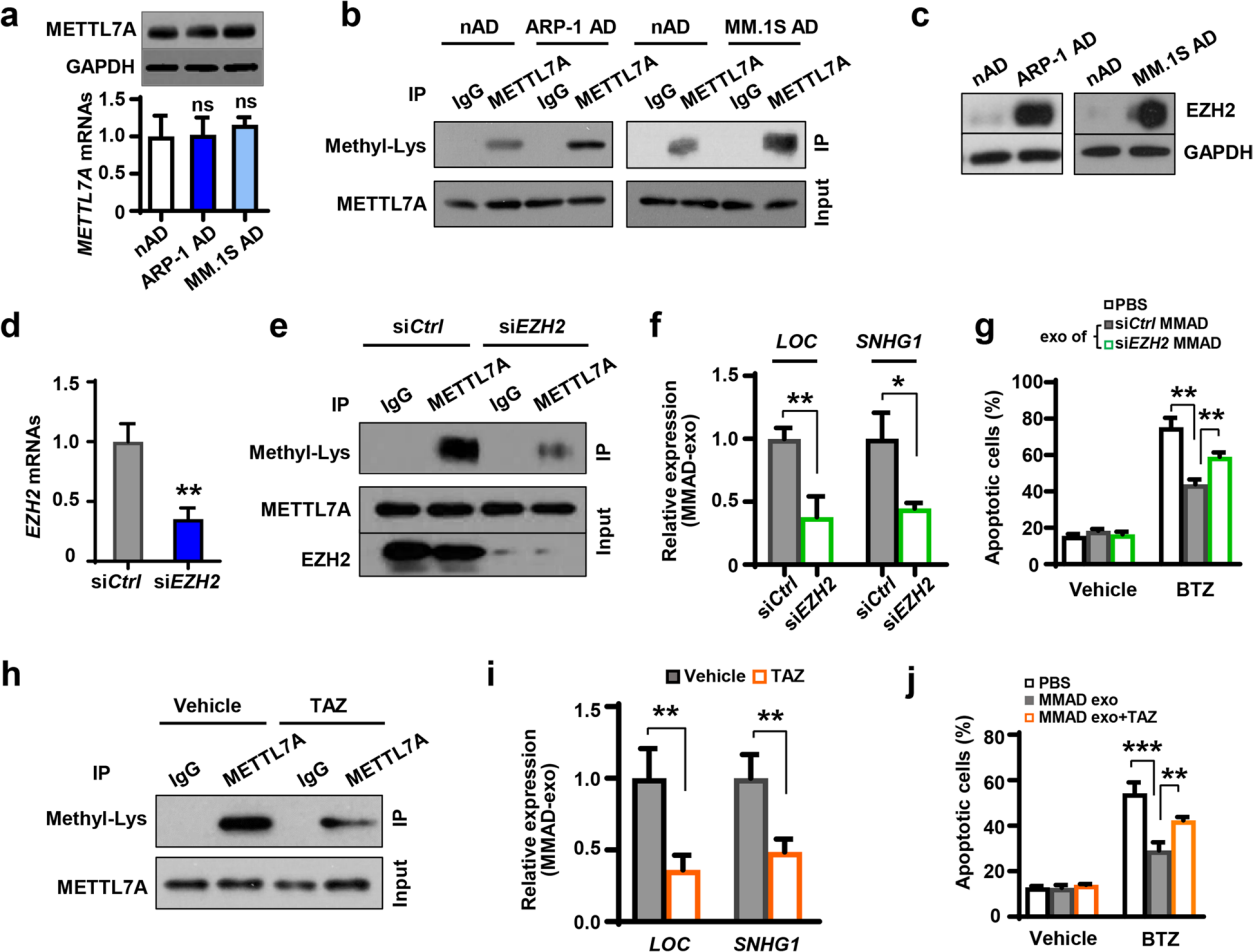
MM cells enhance METTL7A activity through upregulation of EZH2 in adipocytes. **a.** The levels of METTL7A proteins and mRNAs in nADs, ARP-1 treated adipocytes (ARP-1 AD), and MM.1S treated adipocytes (MM.1S AD). **b.** Shown is the lysine methylation (Methyl-Lys) level in the lysates of nADs, ARP-1 ADs, and MM.1S ADs immunoprecipitated with an anti-METTL7A resin. Cell lysates immunoprecipitated with IgG and METTL7A level in the input served as control. **c.** Western blotting shows the level of EZH2 in nADs, ARP-1 ADs, and MM.1S ADs. **d.** Shown is the efficiency of EZH2 siRNA transfection in adipocytes exposed to ARP-1 cells (MMADs). **e.** Shown is the level of Methyl-Lys in the lysates of si*Ctrl* or si*EZH2* MMADs immunoprecipitated with anti-METTL7A resin. Cell lysates immunoprecipitated with IgG and METTL7A and EZH2 levels in the input served as control. **f.** Quantitative real-time PCR analysis shows the relative expression of exosomal *LOC606724 (LOC)* and *SNHG1* derived from MMADs carrying si*Ctrl* or si*EZH2*. **g.** Annexin V-binding assay shows the percentages of apoptotic ARP-1 cells treated with 5 nM bortezomib (BTZ) and exosomes derived from MMADs carrying si*Ctrl* or si*EZH2* for 24 h. **h-j.** MMADs were treated with or without 100 nM tazemetostat (TAZ) for 24 h. **h.** Shown is the Methyl-Lys level in the lysates of TAZ-treated MMADs immunoprecipitated with anti-METTL7A resin. Cell lysates immunoprecipitated with IgG and METTL7A level in the input served as control. **i.** Quantitative real-time PCR analysis shows the relative expression of exosomal *LOC* and *SNHG1* derived from MMADs treated with or without TAZ. **j.** Annexin V-binding assay shows the percentages of apoptotic ARP-1 cells treated with 5 nM BTZ and exosomes derived from MMADs treated with or without TAZ. *P < 0.05; **P < 0.01; ***P < 0.001. *P* values were determined by Student *t*-tests for comparison of two groups and one-way ANOVA for comparison of more than two groups

Since the activity of METTL7A is higher in MMADs than nADs, we considered how MM cells could regulate METTL7A in adipocytes. We exposed nADs to MM ARP-1 or MM.1S cells, but did not observe obvious changes in the levels of METTL7A mRNA and protein in adipocytes (Fig. 7a), suggesting that MM cells do not affect adipocyte METTL7A expression. We next investigated post-translational modification, where one of the key mechanisms is protein methylation [36]. By pulling down the lysates of adipocytes with anti-METTL7A antibodies, we detected higher methylation levels on METTL7A protein in the immunoprecipitates of MMADs than in the immunoprecipitates of nADs (Fig. 7b), implicating that MM cells enhance METTL7A protein methylation. In addition, EZH2, a lysine methyltransferase that can methylate both histone and non-histone proteins, was expressed at a higher level in MMADs than in nADs (Fig. 7c) [12]. We considered if EZH2 could directly mediate METTL7A protein methylation. Indeed, knocking down EZH2 expression in MMADs using the specific siRNA (si*EZH2*) (Fig. 7d) remarkably reduced the methylation level on METTL7A protein when compared to that in si*Ctrl* MMADs (Fig. 7e). The levels of exosomal *LOC606724* or *SNHG1* dropped significantly in si*EZH2* MMADs than in si*Ctrl* MMADs (Fig. 7f). Compared to exosomes from si*Ctrl* MMADs, the protective effect offered by the exosomes of si*EZH2* MMADs was attenuated as more MM cells died from apoptosis after bortezomib treatment (Fig. 7g). Alternatively, we used EZH2 inhibitor, tazemetostat [37], to attenuate its methyltransferase activity, and we found similar results from the genetic knockdowns. Treatment of MMADs with tazemetostat significantly reduced the levels of lysine methylation on METTL7A protein and exosomal *LOC606724* or *SNHG1*, compared to those treated with vehicle control (Fig. 7h and i). Not surprisingly, addition of tazemetostat weakened the protective effect of MMAD exosomes given to bortezomib-treated MM cells (Fig. 7j).

**Fig. 8:**
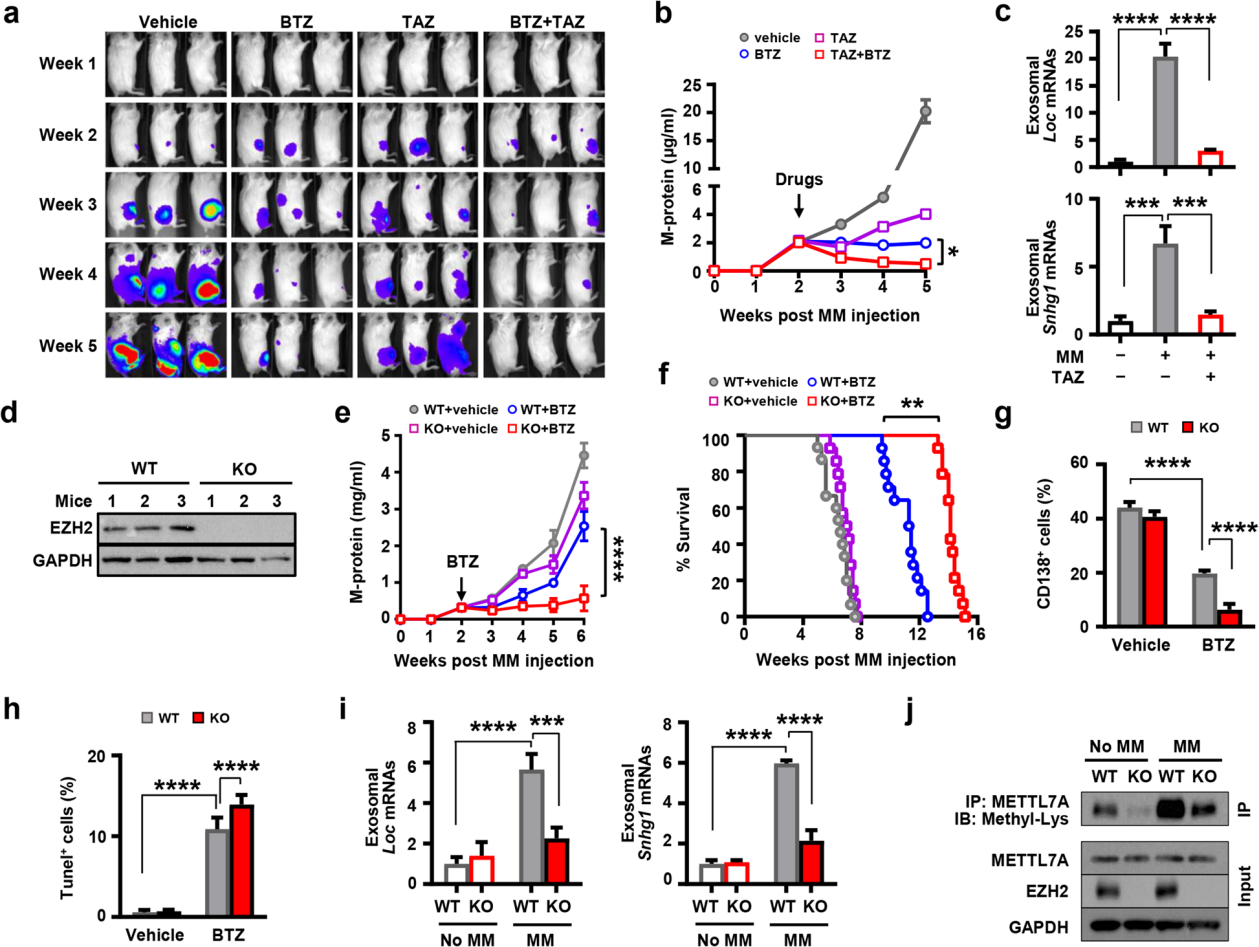
Depletion of EZH2 lowers adipocyte exosomal LncRNA levels and improves the therapeutic efficacy of bortezomib in the MM mouse models. **a-c.** NSG mice were injected intrafemorally with 5 × 10^5^ luciferase-carrying ARP-1 MM cells. After 2 weeks, mice were treated with bortezomib (BTZ, 0.5 mg/kg) alone or in combination with tazemetostat (TAZ, 0.5 g/kg). Treatment with vehicle served as control. Shown are representative bioluminescent images (**a**) and serum M-protein levels (**b**) from week 0 to week 5. **c.** Quantitative real-time PCR analysis shows the relative expression of exosomal *Loc* and *Snhg1* derived from adipocytes isolated from bone marrow of mice bearing with MM cells at week 5. Data shown as mean ± SD (n = 3 mice/group). **d-h**. Wild type (WT) or adipocyte-specific EZH2 knockout (KO) mice intrafemorally injected with murine MM Vk12598 cells (1 × 10^6^ cells/mouse) were treated intraperitoneally with BTZ (0.5 mg/kg) 2 weeks after MM cell injection. **d.** Western blotting shows the levels of EZH2 protein in marrow adipocytes of WT and EZH2 KO mice. GAPDH served as loading control. **e**–**h**. Shown are serum M-proteins levels (**e**), Kaplan–Meier analysis on survival (**f**), percentages of marrow CD138^+^ (**g**), and TUNEL^+^ MM cells (**h**) in WT or KO mice with or without treatment of BTZ. **i**-**j**. WT or EZH2 KO mice were intrafemorally injected with or without murine MM Vk12598 cells (1 × 10^6^ cells/mouse) for 6 weeks. **i**. Quantitative real-time PCR analysis shows the relative levels of exosomal *Loc* and *Snhg1* derived from adipocytes that were isolated from bone marrow of WT or KO mice at week 6 after MM cell injection. **j**. Shown is the level of Methyl-Lys in the lysates of adipocytes isolated from WT or EZH2 KO mice immunoprecipitated with anti-METTL7A resin. Cell lysates immunoprecipitated with METTL7A and EZH2 levels in the input served as control. Data shown as mean ± SD (n = 4 mice/group). In f, survival curve combined the data from multiple experiments (n = 14–15 mice/group). **P < 0.01; ***P < 0.001; ****P < 0.0001. *P* values were determined by Student *t*-tests for comparison of two groups and one-way ANOVA for comparison of more than two groups

We then examined our in vitro findings in the MM mouse models [11, 12]. In xenografted-mouse model, human MM ARP-1 cells carrying luciferase were intrafemorally injected into NSG mice, and tumor burden was monitored weekly by bioluminescence imaging and serum M-protein levels. Two weeks after ARP-1 cell injection, mice were treated with bortezomib or tazemetostat, alone or in combination. Not surprisingly, treatment of either bortezomib or tazemetostat reduced bioluminescent signals, serum M-protein levels, and combination of two drugs showed synergistical effects (Fig. 8a and b). We isolated the exosomes from the culture medium of adipocytes that were obtained from mouse bone marrow and found that treatment of tazemetostat significantly reduced the levels of *Loc606724* or *Snhg1* in adipocyte exosomes, compared to those treated with the vehicle control (Fig. 8c).

After using tazemetostat for systemic inhibition of EZH2, we established murine MM model in adipocyte-specific EZH2 genetic knockout C57BL/6 mice [12] and determine whether the EZH2 expressed in adipocytes affects MM therapeutic response. We intrafemorally injected murine MM Vk12598 cells into those mice (Fig. 8d) [11, 12]. Two weeks later, Vk12598-bearing mice were treated intraperitoneally with bortezomib or the vehicle control for four weeks. MM tumor burden was monitored by serum M-protein levels. While the treatment of bortezomib reduced M-proteins compared to the vehicle control in wild-type mice (Fig. 8e), the treatment was much more effective in the knockout mice with improved survival (Fig. 8e and f), indicating the functional role of adipocyte EZH2 in MM cell response to chemotherapy. This finding was further confirmed by flow cytometric analysis and TUNEL assay, as more apoptotic CD138^+^ MM cells were observed in the bone marrow of adipocyte-EZH2 knockout mice than those in wild-type mice after bortezomib treatment (Fig. 8g and h). Furthermore, the levels of adipocyte exosomal LncRNAs as well as the methylation level on METTL7A were reduced in MM-bearing EZH2 knockout mice compared to those in MM-bearing wild type mice (Fig. 8i and j). The results from our in vitro and in vivo studies demonstrate an unexplored role of adipocyte exosomal LncRNAs in MM drug resistance and the methyltransferase activity of METTL7A in RNA methylation in adipocytes, where MM cells promote packaging of LncRNAs into adipocyte exosomes by enhancing METTL7A activity through EZH2-mediated METTL7A protein methylation.

## Discussion

In the current study, we have discovered a vicious cycle between MM cells and adipocytes through the regulation of adipocyte exosomal packaging of LncRNAs and its contribution to MM drug resistance. LncRNAs are recognized as an important element in the regulation of gene expression [38], and they participate in various pathological processes of benign and malignant diseases [39–41]. In MM, LncRNA dysfunction is considered an independent predictor of poor survival in patients, indicating its importance to MM development and pathogenesis [27]. We have identified several LncRNAs carried by adipocyte exosomes and associated with MM drug resistance. Their levels are especially higher in patients who did not respond to bortezomib-based therapy than those who responded, and the elevated level is positively correlated to poorer clinical outcomes in MM. By performing gene knock in and down strategies, we determine the importance of two LncRNAs in adipocyte exosome-induced MM drug resistance and elucidate the mechanism of *LOC606724* in MM cell apoptosis since its function in tumors was previously unexplored. Our mechanistic studies demonstrate that *LOC606724* enhances the protein translation of c-Myc through eIF4E by promoting it to bind to c-Myc mRNA, while little or no effect on gene transcription and protein degradation was observed. These findings put a spotlight on *LOC606724* as a new player for regulation of oncogenes, which may play an important role in MM drug resistance. We suspect that other LncRNAs carried by adipocyte exosomes may also offer protective effects against chemotherapy through distinct pathways. Therefore, we believe that the altered profile of LncRNAs in adipocyte exosomes may be useful for predicting MM therapeutic response in patients.

For the first time here, we demonstrate that LncRNAs are enriched into adipocyte exosomes upon methylation. For example, the levels of methylated *LOC606724* and *SNHG1* in MMADs are especially high comparing to those in nADs, and the methylated LncRNAs have a higher activity to interact with RNA binding proteins, leading to an increased transfer of LncRNAs into the exosomes. We investigated the mechanism of how LncRNAs are methylated, focusing on m^6^A methylation, shown to be the most common in regulation of RNA metabolism. The RNA m^6^A methylation can be activated by methyltransferases, and several METTL family members have been identified with m^6^A methyltransferase activity [34, 35]. While we failed to pull down the known methyltransferases by *LOC606724*, we have identified METTL7A protein in the immunoprecipitates. As a membrane protein, METTL7A anchors into the endoplasmic reticulum membrane and recruits cellular proteins to form lipid droplets [31]. Although its function in cancers is rarely investigated, previous studies show that METTL7A may be associated with the development of thyroid cancer [42]. Our study is the first to demonstrate that METTL7A possesses methyltransferase activity, participates in LncRNA m^6^A methylation, and contributes to adipocyte-induced MM drug resistance. In addition, we determine the region of 76–172 aa in METTL7A protein mostly responsible for RNA m^6^A methyltransferase activity and the position of the methylation motif on *LOC606724*. We further discover that METTL7A-activated RNA m^6^A methylation contributes to LncRNA interaction with RNA binding protein. These findings may explain why LncRNAs are enriched into exosomes.

We also investigate how MM cells regulate the activity of METTL7A in RNA methylation, focusing on EZH2-mediated protein methylation. EZH2 acts as a histone-lysine N-methyltransferase enzyme and has activity in the methylation of non-histone proteins. Our previous studies have demonstrated that upregulation of EZH2 is the key for MM-induced adipocyte reprogramming [12]. In this study, we show that pharmaceutical or genetical depletion of EZH2 reduces METTL7A protein methylation levels in MMADs and impairs the effects of METTL7A on LncRNA m^6^A methylation and exosomal LncRNA package. Of note, the EZH2 inhibitor tazemetostat has been approved by U.S. Food and Drug Administration for the treatment of follicular lymphoma [43]. In MM, preclinical studies show that tazemetostat has a tumoricidal activity with additional therapeutic effect on MM-associated bone disease through regulation of osteoblast differentiation and activity [44]. In line with other reports, we confirm that combination of bortezomib with tazemetostat has a synergistic effect on reduction of tumor growth in the MM mouse model. More importantly, treatment of tazemetostat significantly reduces exosomal LncRNA levels in marrow adipocytes. Thus, our results may provide an additional mechanistic insight for improving the clinical efficacy of tazemetostat within tumor microenvironment.

## Conclusions

In summary, our findings highlight the vicious cycle formed between MM cells and adipocytes mediated by adipocyte-secreted exosomes, in which adipocytes affect MM cell response to therapies and in turns MM cells educate adipocytes through the EZH2/METTL7A/LncRNA axis.

## Availability of supporting data

Data needed to evaluate the conclusions in the paper are provided in the main text or the Supplementary Materials. Stable cell lines carrying targeted shRNA are available through establishment of Material Transfer Agreement between Houston Methodist Research Institute and request institution.

## Supplementary Information

**Additional file 1: Figure S1.** Confocal microscopy shows the internalization of exosomes into MM cells. **Figure S2**. Representative Annexin V analysis of MM cells that were treated with therapeutic drugs and adipocyte exosomes.** Table S1.** Primers used in the ORF or expression of His-tagged METTL7A.** Table S2.** Primers used in construct shRNAs.** Table S3. **Custom RNA oligonucleotides containing putative METTL7A binding site on the *LOC* transcript.** Table S4.** Primers used in quantitative real-time PCR analysis.

## Abbreviations

CD: Cluster of differentiation
Cyt c: Cytochrome c
ELISA: Enzyme-linked immunosorbent assay
EZH2: Enhancer of zeste homolog 2
HSP90: Heat shock protein 90
hnRNPA2B1: Heterogeneous Nuclear Ribonucleoprotein A2/B1
hnRNPU: Heterogeneous nuclear ribonucleoprotein U
IRF-4: Interferon regulatory factor 4
LncRNA: Long non-coding RNAs
LOC: LOC606724
m6A: N6-methyladenosine
MAF: Avian Musculoaponeurotic Fibrosarcoma
MeRIP-seq: Methylated RNA immunoprecipitation sequencing
METTL7A: Methyltransferase like 7A
MM: Multiple myeloma
MMAD: MM-associated adipocyte
MMRF: Multiple Myeloma Research Foundation
MSC: Mesenchymal stem cell
nAD: Normal adipocytes
NSG: NOD-scid IL2Rgnull
PTEN: Phosphatase and tensin homolog
Rb: Retinoblastoma protein
RIP: RNA immunoprecipitation
shRNA: Short hairpin RNA
siRNA: Small interfering RNA
SNHG1: Small nucleolar RNA host gene 1
SRAMP: Sequence-based RNA adenosine methylation site predictor

## References

[1] Kumar SK, Rajkumar V, Kyle RA, van Duin M, Sonneveld P, Mateos MV, Gay F, Anderson KC (2017). Multiple myeloma Nat Rev Dis Primers.

[2] Kyle RA, Rajkumar SV (2004). Multiple myeloma. N Engl J Med.

[3] Rajkumar SV, Dimopoulos MA, Palumbo A, Blade J, Merlini G, Mateos MV, Kumar S, Hillengass J, Kastritis E, Richardson P (2014). International Myeloma Working Group updated criteria for the diagnosis of multiple myeloma. Lancet Oncol.

[4] Moreau P, Richardson PG, Cavo M, Orlowski RZ, San Miguel JF, Palumbo A, Harousseau JL (2012). Proteasome inhibitors in multiple myeloma: 10 years later. Blood.

[5] Robak P, Drozdz I, Szemraj J, Robak T (2018). Drug resistance in multiple myeloma. Cancer Treat Rev.

[6] Gimble JM, Robinson CE, Wu X, Kelly KA (1996). The function of adipocytes in the bone marrow stroma: an update. Bone.

[7] Meunier P, Aaron J, Edouard C, Vignon G (1971). Osteoporosis and the replacement of cell populations of the marrow by adipose tissue. A quantitative study of 84 iliac bone biopsies. Clin Orthop Relat Res.

[8] Morris EV, Edwards CM (2016). Bone Marrow Adipose Tissue: A New Player in Cancer Metastasis to Bone. Front Endocrinol (Lausanne).

[9] Nieman KM, Kenny HA, Penicka CV, Ladanyi A, Buell-Gutbrod R, Zillhardt MR, Romero IL, Carey MS, Mills GB, Hotamisligil GS (2011). Adipocytes promote ovarian cancer metastasis and provide energy for rapid tumor growth. Nat Med.

[10] Fairfield H, Dudakovic A, Khatib CM, Farrell M, Costa S, Falank C, Hinge M, Murphy CS, DeMambro V, Pettitt JA (2021). Myeloma-Modified Adipocytes Exhibit Metabolic Dysfunction and a Senescence-Associated Secretory Phenotype. Cancer Res.

[11] Li Z, Liu H, He J, Wang Z, Yin Z, You G, Wang Z, Davis RE, Lin P, Bergsagel PL (2021). Acetyl-CoA Synthetase 2: A Critical Linkage in Obesity-Induced Tumorigenesis in Myeloma. Cell Metab.

[12] Liu H, He J, Koh SP, Zhong Y, Liu Z, Wang Z, Zhang Y, Li Z, Tam BT, Lin P (2019). Reprogrammed marrow adipocytes contribute to myeloma-induced bone disease. Sci Transl Med.

[13] Liu Z, Xu J, He J, Liu H, Lin P, Wan X, Navone NM, Tong Q, Kwak LW, Orlowski RZ, Yang J (2015). Mature adipocytes in bone marrow protect myeloma cells against chemotherapy through autophagy activation. Oncotarget.

[14] Trotter TN, Gibson JT, Sherpa TL, Gowda PS, Peker D, Yang Y (2016). Adipocyte-Lineage Cells Support Growth and Dissemination of Multiple Myeloma in Bone. Am J Pathol.

[15] Caers J, Deleu S, Belaid Z, De Raeve H, Van Valckenborgh E, De Bruyne E, Defresne MP, Van Riet I, Van Camp B, Vanderkerken K (2007). Neighboring adipocytes participate in the bone marrow microenvironment of multiple myeloma cells. Leukemia.

[16] Hou J, Wei R, Qian J, Wang R, Fan Z, Gu C, Yang Y (2019). The impact of the bone marrow microenvironment on multiple myeloma (Review). Oncol Rep.

[17] O'Brien K, Breyne K, Ughetto S, Laurent LC, Breakefield XO (2020). RNA delivery by extracellular vesicles in mammalian cells and its applications. Nat Rev Mol Cell Biol.

[18] Sun Z, Yang S, Zhou Q, Wang G, Song J, Li Z, Zhang Z, Xu J, Xia K, Chang Y (2018). Emerging role of exosome-derived long non-coding RNAs in tumor microenvironment. Mol Cancer.

[19] Gezer U, Ozgur E, Cetinkaya M, Isin M, Dalay N (2014). Long non-coding RNAs with low expression levels in cells are enriched in secreted exosomes. Cell Biol Int.

[20] Chesi M, Matthews GM, Garbitt VM, Palmer SE, Shortt J, Lefebure M, Stewart AK, Johnstone RW, Bergsagel PL (2012). Drug response in a genetically engineered mouse model of multiple myeloma is predictive of clinical efficacy. Blood.

[21] Mitry RR, Hughes RD: *Human Cell Culture Protocols.* Humana Press; 2011.

[22] He J, Liu Z, Zheng Y, Qian J, Li H, Lu Y, Xu J, Hong B, Zhang M, Lin P (2012). p38 MAPK in myeloma cells regulates osteoclast and osteoblast activity and induces bone destruction. Cancer Res.

[23] Liu H, Liu Z, Du J, He J, Lin P, Amini B, Starbuck MW, Novane N, Shah JJ, Davis RE (2016). Thymidine phosphorylase exerts complex effects on bone resorption and formation in myeloma. Sci Transl Med.

[24] Khalil AM, Guttman M, Huarte M, Garber M, Raj A, Rivea Morales D, Thomas K, Presser A, Bernstein BE, van Oudenaarden A (2009). Many human large intergenic noncoding RNAs associate with chromatin-modifying complexes and affect gene expression. Proc Natl Acad Sci U S A.

[25] Jeppesen DK, Fenix AM, Franklin JL, Higginbotha JN, Zhang Q, Zimmerman LJ, Liebler DC, Ping J, Liu Q, Evans R (2019). Reassessment of Exosome Composition. Cell.

[26] Qu L, Ding J, Chen C, Wu ZJ, Liu B, Gao Y, Chen W, Liu F, Sun W, Li XF (2016). Exosome-Transmitted lncARSR Promotes Sunitinib Resistance in Renal Cancer by Acting as a Competing Endogenous RNA. Cancer Cell.

[27] Amodio N, Stamato MA, Juli G, Morelli E, Fulciniti M, Manzoni M, Taiana E, Agnelli L, Cantafio MEG, Romeo E (2018). Drugging the lncRNA MALAT1 via LNA gapmeR ASO inhibits gene expression of proteasome subunits and triggers anti-multiple myeloma activity. Leukemia.

[28] Zhan F, Barlogie B, Arzoumanian V, Huang Y, Williams DR, Hollmig K, Pineda-Roman M, Tricot G, van Rhee F, Zangari M (2007). Gene-expression signature of benign monoclonal gammopathy evident in multiple myeloma is linked to good prognosis. Blood.

[29] Zietzer A, Hosen MR, Wang H, Goody PR, Sylvester M, Latz E, Nickenig G, Werner N, Jansen F (2020). The RNA-binding protein hnRNPU regulates the sorting of microRNA-30c-5p into large extracellular vesicles. J Extracell Vesicles.

[30] He RZ, Jiang J, Luo DX (2020). The functions of N6-methyladenosine modification in lncRNAs. Genes Dis.

[31] Zehmer JK, Bartz R, Liu P, Anderson RG (2008). Identification of a novel N-terminal hydrophobic sequence that targets proteins to lipid droplets. J Cell Sci.

[32] Ignatova VV, Jansen P, Baltissen MP, Vermeulen M, Schneider R (2019). The interactome of a family of potential methyltransferases in HeLa cells. Sci Rep.

[33] van Tran N, Ernst FGM, Hawley BR, Zorbas C, Ulryck N, Hackert P, Bohnsack KE, Bohnsack MT, Jaffrey SR, Graille M, Lafontaine DLJ (2019). The human 18S rRNA m6A methyltransferase METTL5 is stabilized by TRMT112. Nucleic Acids Res.

[34] Pendleton KE, Chen B, Liu K, Hunter OV, Xie Y, Tu BP, Conrad NK (2017). The U6 snRNA m(6)A Methyltransferase METTL16 Regulates SAM Synthetase Intron Retention. Cell.

[35] Liu J, Yue Y, Han D, Wang X, Fu Y, Zhang L, Jia G, Yu M, Lu Z, Deng X (2014). A METTL3-METTL14 complex mediates mammalian nuclear RNA N6-adenosine methylation. Nat Chem Biol.

[36] Han D, Huang M, Wang T, Li Z, Chen Y, Liu C, Lei Z, Chu X (2019). Lysine methylation of transcription factors in cancer. Cell Death Dis.

[37] Italiano A, Soria JC, Toulmonde M, Michot JM, Lucchesi C, Varga A, Coindre JM, Blakemore SJ, Clawson A, Suttle B (2018). Tazemetostat, an EZH2 inhibitor, in relapsed or refractory B-cell non-Hodgkin lymphoma and advanced solid tumours: a first-in-human, open-label, phase 1 study. Lancet Oncol.

[38] Kopp F, Mendell JT (2018). Functional Classification and Experimental Dissection of Long Noncoding RNAs. Cell.

[39] Bartolomei MS, Zemel S, Tilghman SM (1991). Parental imprinting of the mouse H19 gene. Nature.

[40] Goodrich JA, Kugel JF (2006). Non-coding-RNA regulators of RNA polymerase II transcription. Nat Rev Mol Cell Biol.

[41] Zheng J, Huang X, Tan W, Yu D, Du Z, Chang J, Wei L, Han Y, Wang C, Che X (2016). Pancreatic cancer risk variant in LINC00673 creates a miR-1231 binding site and interferes with PTPN11 degradation. Nat Genet.

[42] Zhou S, Shen Y, Zheng M, Wang L, Che R, Hu W, Li P (2017). DNA methylation of METTL7A gene body regulates its transcriptional level in thyroid cancer. Oncotarget.

[43] Hoy SM (2020). Tazemetostat: First Approval. Drugs.

[44] Gulati N, Beguelin W, Giulino-Roth L (2018). Enhancer of zeste homolog 2 (EZH2) inhibitors. Leuk Lymphoma.

