# Supplementary material for: Induction of m6A methylation in adipocyte exosomal LncRNAs mediates myeloma drug resistance
Source: J Exp Clin Cancer Res. 2022 Jan 3;41:4. doi: 10.1186/s13046-021-02209-w (PMC8722039; doi:10.1186/s13046-021-02209-w)
Supplement: Supplementary file 1 — Additional file 1: Figure S1. Confocal microscopy shows the internalization of exosomes into MM cells. Figure S2. Representative Annexin V analysis of MM cells that were treated with therapeutic drugs and adipocyte exosomes. Table S1. Primers used in the ORF or expression of His-tagged METTL7A. Table S2. Primers used in construct shRNAs. Table S3. Custom RNA oligonucleotides containing putative METTL7A binding site on the LOC transcript. Table S4. Primers used in quantitative real-time PCR analysis. [file 13046_2021_2209_MOESM1_ESM.docx]

**Supplementary Materials**

**I****nduction of m^6^A methylation in adipocyte exosomal LncRNAs mediates myeloma drug resistance**

Zhiming Wang^1^, Jin He^1^, Duc-hiep Bach^1^, Yung-hsing Huang^1^, Zongwei Li^1^, Huan Liu^2^, Pei Lin^3^, and Jing Yang^1*^

**Supplementary Figures**

**Supplementary Tables**

**Supplementary Figures**


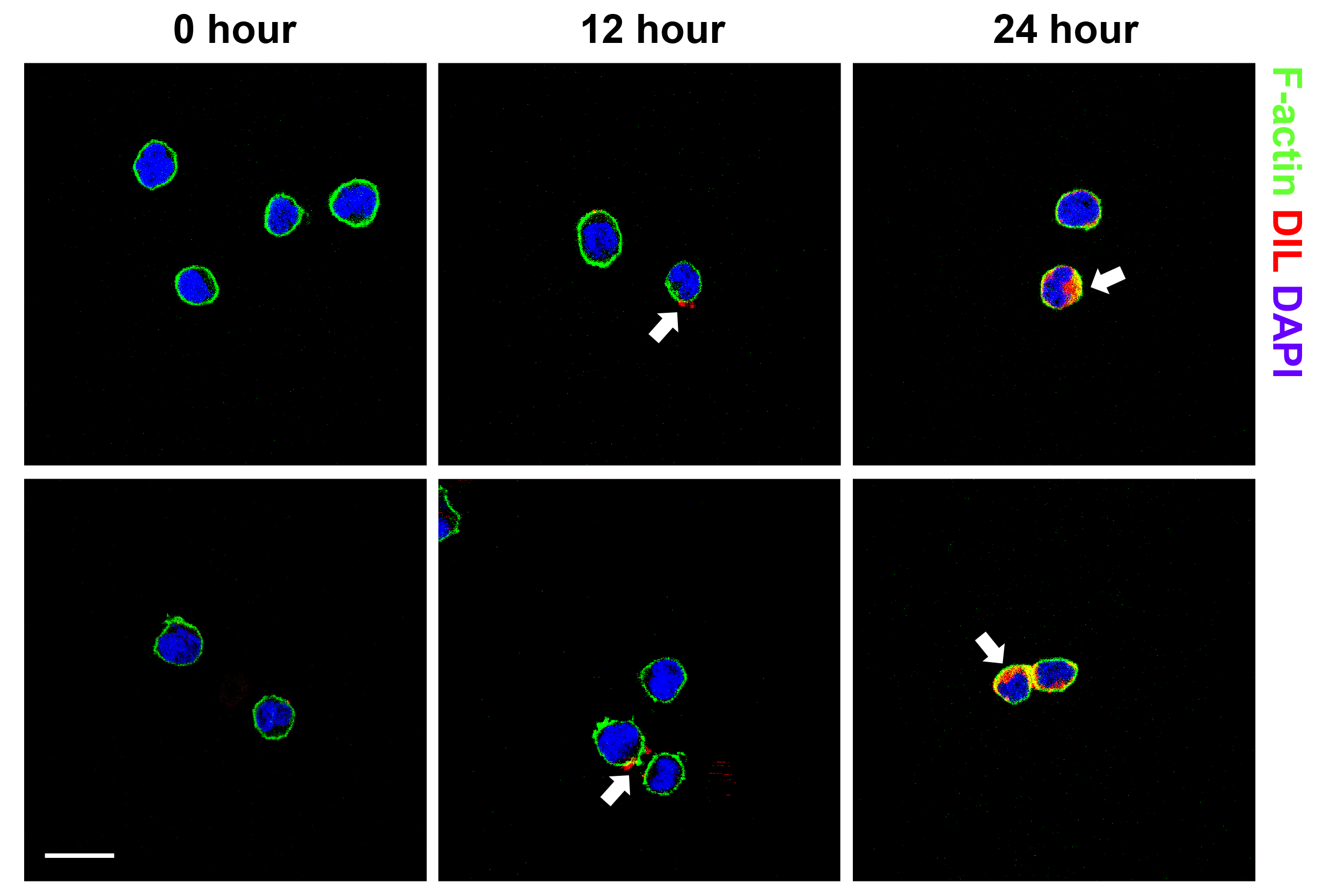


**Supplementary Figure 1. Confocal microscopy shows the internalization of exosomes into MM cells.** ARP-1 cells were exposed to Dil pre-stained adipocyte-derived exosomes and then stained with Alexa Fluor 488 Phalloidin for F-actin and DAPI for nucleus staining. Scale bar, 20 μm. Representative images are shown at 0, 12, or 24 h after culturing MM cells with exosomes.

**
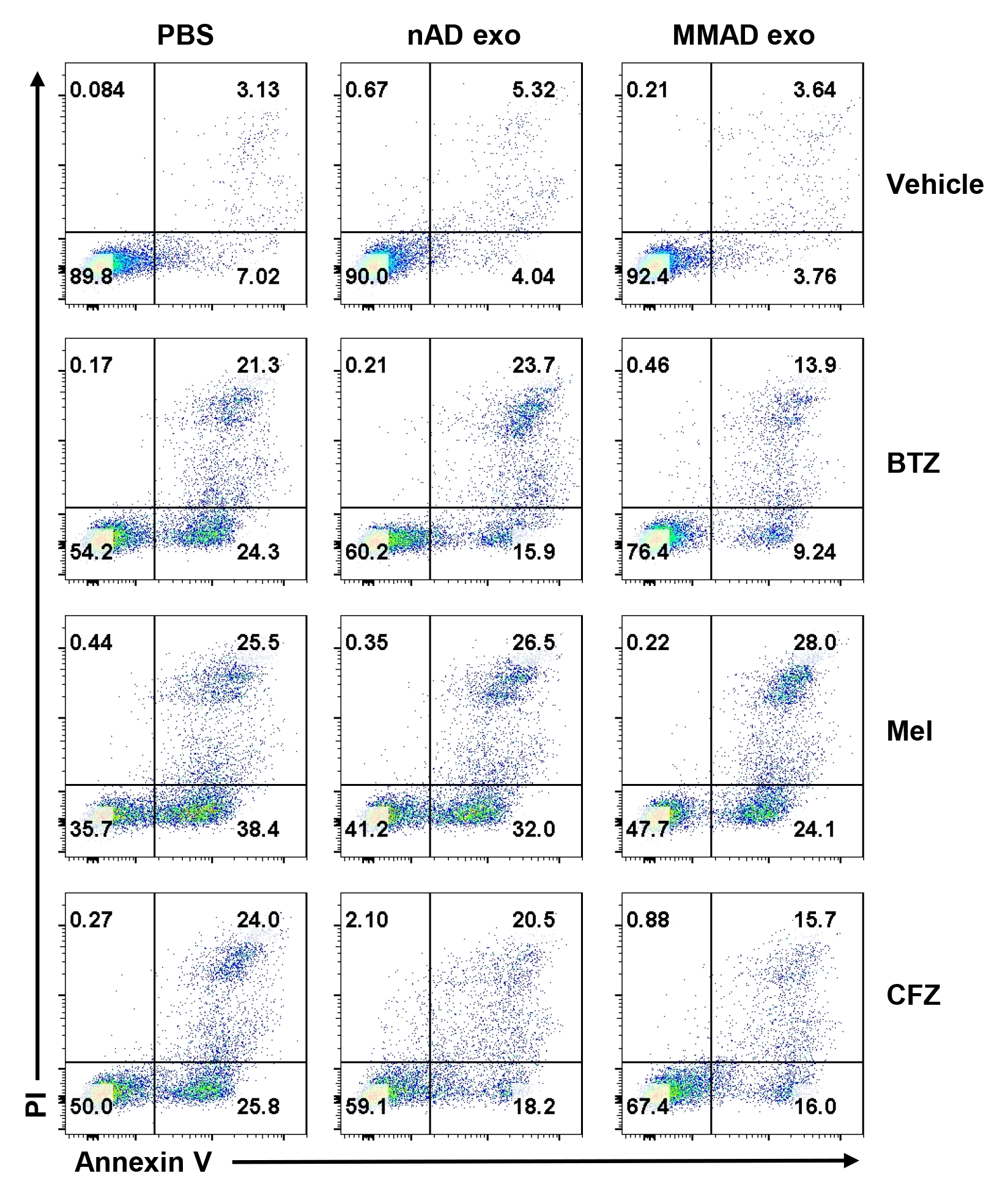
**

**Supplementary Figure 2. Representative Annexin V analysis of MM cells that were treated with therapeutic drugs and adipocyte exosomes.** Representative scatter plots show the percentage of apoptotic MM.1S cells treated with 5 nM bortezomib (BTZ), 25 μM melphalan (Mel), or 25 nM carfilzomib (CFZ) with or without exosomes derived from nADs or MMADs for 24 h. Cells treated with vehicle served as control.

**Supplementary Tables**

**Table S1. Primers used in the ORF or expression of His-tagged METTL7A.**

| Gene | Forward | Reverse | Vector |
| --- | --- | --- | --- |
| *LOC*  (sense*)* | CCGCTCGAGGTAATCCTGAGGCCTCAGAAC | CCGGAATTCACATCAGCATGGCTGGTTTCT | pcDNA3.1 (-) |
| *LOC*  (antisense) | CCGCTCGAGACATCAGCATGGCTGGTTTCT | CCGGAATTCGTAATCCTGAGGCCTCAGAAC | pcDNA3.1 (-) |
| *SNHG1* | CCGCTCGAGCTCATTTTTCTACTGCTCG | GCCCTATAGTTTTTTTTTTTTTAAACAGGAC | pcDNA3.1 (-) |
| *METTL7A*  (full length) | CCGGAATTCATGGAGCTTACCATCTTTATC | GCGGCCGCTTTCACAGCATATCCATAG | pET-28a (+) |
| *METTL7A*  (Δ1) | GAATTCATGGAGCTTACCATCTT | GCGGCCGCCAGCAGGGAGAGTTTCCCG | pET-28a (+) |
| *METTL7A*  (Δ2) | GaattcATGGAGCTTACCATCTT | GCGGCCGCGAAATAGAAAGCCCCTC | pET-28a (+) |

**Table S2. Primers used in construct shRNAs.**

| shRNA | Forward | Reverse |
| --- | --- | --- |
| sh*Ctrl* | CCGGCCTAAGGTTAAGTCGCCCTCGCTCGAGCGAGGGCGACTTAACCTTAGGTTTTTG | AATTCAAAAACCTAAGGTTAAGTCGCCCTCGCTCGAGCGAGGGCGACTTAACCTTAGG |
| sh*LOC606724* | CCGGGGTACCTCCCAGACTCTGATGCTCGAGCATCAGAGTCTGGGAGGTACCTTTTTG | AATTCAAAAAGGTACCTCCCAGACTCTGATGCTCGAGCATCAGAGTCTGGGAGGTACC |
| sh*SNHG1* | CCGGGGTTTGCTGTGTATCACATTTCTCGAGAAATGTGATACACAGCAAACCTTTTTG | AATTCAAAAAGGTTTGCTGTGTATCACATTTCTCGAGAAATGTGATACACAGCAAACC |

**Table S3. Custom RNA oligonucleotides containing putative METTL7A binding site on the *LOC* transcript**

| Custom RNA oligonucleotides | Sequences |
| --- | --- |
| M1 | CCAGAAGCGCUUGGACAGGCUGGAGGAG |
| M2 | GGAGGAGACAGUCCAGGCC |
| M3 | GGUACCUCCCAGACUCUGAUGACUGGU |
| M4 | UCUGAUGACUGGUCCCCUAGACACG |
| M5 | CACCGCGCUGAGGGGACUGACCCAUGGCAGGG |
| M6 | CCCCCUGACUUACUACAUCA |
| M7 | CACAGGAACAGGGCCUUCGUGG |
| M5AΔG | CACCGCGCUGAGGGGGCUGACCCAUGGCAGGG |

**Table S4. Primers used in quantitative real-time PCR analysis.**

| Gene | Forward | Reverse |
| --- | --- | --- |
| *LOC606724* | GTTTGCTGAACCAACCAGCC | TGGTTTCTACCCACGAAGGC |
| *GAPDH* | CTGGGCTACACTGAGCACC | AAGTGGTCGTTGAGGGCAATG |
| *BCAR4* | TGGTGGCTATGGAGTTCTGC | TCTTGCCTTGGGGACAGTTC |
| *C14orf167* | GCCGATCCTGTGAGCAGAAA | ATTCAAACCCGGGCAGTCTT |
| *c-Myc* | AATGAAAAGGCCCCCAAGGTAGTTATCC | GTCGTTTCCGCAACAAGTCCTCTTC |
| *EIF4E* | TGGCGACTGTCGAACCG | AGATTCCGTTTTCTCCTCTTCTGTAG |
| *EZH2* | GGACCACAGTGTTACCAGCAT | GTGGGGTCTTTATCCGCTCAG |
| *HLA-H* | GACGGGTCCTTCTTCCTTGA | GACTTTAGAACCGGGACCGC |
| *HnRNPA2B1* | AGCTTTGAAACCACAGAAGAA | TTGATCTTTTGCTTGCAGGA |
| *KIAA0114* | ACTCAATGCAGCTGACCCTT | GTGACCTTGCTATAGCGCCT |
| *LOC407835* | CCAACAGCGGTTACGGGAT | TTAGGAGGCGGCTCTTTCAC |
| *LOC728855* | ATGTTCAAGGGTGGCCCAAA | TCTCCTGATACAGGCGGGAT |
| *NCRNA00219* | GCCCTGCCCCCGTATAAAAT | TAGGTGATCACTCCCGACGA |
| *PIPSL* | GGTGTTCACCTTGGTTGTCC | ACTCACTGTTGTCCACACTTG |
| *PTTG3P* | ACGAAGAACCAGGCATCCTT | TGGGAGCATCGAATGTTTTGC |
| *RASA4P* | GAACTTTGGCAGCTCGCAC | TTGTCCCCACAGAAGATGCC |
| *RPL13L* | ACTCAGCTGACAGGACCAGA | TATATGCCGAAGAGCTGGGC |
| *SNHG1* | CTGTTCCCGTCATGAGCCTT | GCAAGGCCCTGAATGAGCTA |
| *SNHG6* | CTGCGAGGTGCAAGAAAGC | TCAATACATGCCGCGTGATC |
| *SNHG9* | GGGAATCCACCCGAAGAGTG | TGGGAGGACCAGTGTCCTAA |
| *TUG1* | AGCGTGGGTGTACGTAAAGG | CCAAGGATTGGGGAACTGCT |
| *METTL7A* | GCGGGAGCTCTTCAGTAACC | TCTCAAAGTTGGGGTTGGGG |
| *Loc606724 (Loc)* | AGCTCGGATACCGTGTCAAG | GATGAGGGCTCTGGGGTTCT |
| *Snhg1* | TCCTTGTTCGGGGTTTGAGG | ACAGCACCCTGACTACAAGC |
| *Gapdh* | AGGTCGGTGTGAACGGATTTG | TGTAGACCATGTAGTTGAGGTCA |
